# Plant-Derived Bioactive Compounds, Cancer Stemness and Cellular Senescence: A Narrative Review of Preclinical Evidence and Translational Relevance to Cancer Recurrence and Drug Resistance

**DOI:** 10.3390/nu18213522

**Published:** 2026-10-26

**Authors:** Sara Naseer, Wai-Yann Tan, Yin-Teng Sow, Zhi-Yin Wai, Sahana-Shri Ramanitharan, Paey-Ern Bay, Ching-Hsein Chen, Soi-Moi Chye

**Affiliations:** 1 School of Health Science, IMU University, No. 126, Jalan Jalil Perkasa 19, Bukit Jalil, Kuala Lumpur 57000, Malaysia; 00000045999@student.imu.edu.my (S.N.); 00000045469@student.imu.edu.my (W.-Y.T.); 00000045914@student.imu.edu.my (Y.-T.S.); 00000045436@student.imu.edu.my (Z.-Y.W.); 00000045165@student.imu.edu.my (S.-S.R.); 00000045365@student.imu.edu.my (P.-E.B.); 2 Department of Microbiology, Immunology and Biopharmaceuticals, College of Life Sciences, A25-303 Room, Life Sciences Hall, No. 300, Syuefu Road, National Chiayi University, Chiayi City 600355, Taiwan; 3 School of Health Science, Division of Applied Biomedical Science and Biotechnology, IMU University, No. 126, Jalan Jalil Perkasa 19, Bukit Jalil, Kuala Lumpur 57000, Malaysia

**Keywords:** cancer stem cells, cellular senescence, senescence-associated secretory phenotype, plant-derived bioactive compounds, cancer recurrence, drug resistance, therapy-induced senescence

## Abstract

Background/Objectives: Cancer recurrence and drug resistance arise from tumour-cell plasticity and microenvironmental adaptations that may persist after treatment. Cancer stem cells (CSCs) are associated with self-renewal, tumour initiation, residual disease, and treatment tolerance, whereas cellular senescence has context-dependent effects. Although therapy-induced senescence may restrict tumour-cell proliferation, persistent senescent tumour or stromal cells and their senescence-associated secretory phenotype (SASP) can, in selected models, promote inflammatory signalling, stemness-associated phenotypes and therapeutic resistance. This narrative review critically evaluates preclinical and translational evidence concerning curcumin, resveratrol, epigallocatechin gallate, berberine, piperine and shikonin as potential adjunctive modulators of CSCs and senescence-associated processes. Methods: Relevant literature was identified through searches of the MEDLINE/PubMed database from June 2000 and July 2026, using terms related to plant-derived bioactive compounds, CSCs, cellular senescence, SASP, recurrence and drug resistance. Evidence was evaluated according to model relevance, exposure, functional validation and whether it represented direct cross-axis evidence, intermediate mechanistic evidence, or marker- and pathway-level support. Results: Most studies examined CSCs and senescence as separate processes. Direct evidence showing that the same compound, within the same cancer model, modifies a validated cancer stem-cell function and a defined senescence or SASP and subsequently reduces resistance or recurrence is limited or absent. Moreover, poor bioavailability, variable formulations, potentially unachievable experimental exposures, drug interactions, tumour heterogeneity and limited clinical evidence constrain translation. Conclusions: Future studies should incorporate pharmacologically relevant exposures, functional and longitudinal endpoints, causal validation and biomarker-guided combination designs to determine whether these compounds can safely complement standard anticancer therapies.

## 1. Introduction

Cancer remains a major global health burden despite substantial advances in surgery, radiotherapy, systemic treatment, molecularly targeted agents, and immunotherapy. The most clinically consequential challenge is not merely initial tumour control but the persistence or re-emergence of disease after treatment. Cancer recurrence may arise from residual malignant cells that survive therapy, adapt to selective pressure, and subsequently repopulate the tumour and metastatic niches [1,2].

Drug resistance is similarly multifactorial. Intrinsic and acquired resistance can be shaped by tumour heterogeneity, clonal evolution, altered drug transport and metabolism, DNA-damage repair, cell-state plasticity, microenvironmental protection, and immune escape. These processes frequently coexist rather than act in isolation, helping to explain why an early therapeutic response may not translate into durable disease control [3].

### 1.1. Cancer Stemness as a Contributor to Persistence, Relapse, and Resistance

Cancer stem cells (CSCs) are best understood as a functional and plastic cell state rather than as a uniformly fixed, rare cell population. In selected cancer models, CSC-associated populations can sustain self-renewal, tumour initiation, metastatic competence, adaptation to stress, and resistance to treatment. Their phenotype is influenced by dynamic interactions with the tumour microenvironment; therefore, marker expression alone is insufficient to establish CSC identity. Cancer-type-appropriate marker combinations must be interpreted together with functional assays, such as sphere formation, clonogenicity, tumour initiation, or treatment sensitisation [2,4].

This concept has important therapeutic implications. Treatments that debulk the proliferating tumour mass may leave behind cells with stemness-associated or reversible drug-tolerant phenotypes. The surviving cells may contribute to minimal residual disease, re-establish intratumoural heterogeneity, and facilitate recurrence. Accordingly, strategies that modulate CSC-related functional properties may complement, rather than replace, standard anticancer therapies [3,4].

### 1.2. Cellular Senescence: A Context-Dependent Response to Cancer Therapy

Cellular senescence is a durable cell-cycle arrest programme that can be induced by telomere dysfunction, oncogenic stress, DNA damage, oxidative stress, and anticancer therapy. In cancer, therapy-induced senescence (TIS) may initially restrain proliferation and can be associated with therapeutic benefit. However, senescence is not defined by a single universal marker, and its biological consequences depend on cell type, inducing stimulus, persistence, and interactions with surrounding immune and stromal cells [5,6].

These distinctions have important therapeutic implications. Senescence induction aims to establish durable tumour-cell arrest, senolysis involves the selective elimination of established senescent cells, and senomorphic intervention modifies the SASP or other features of the senescent phenotype without necessarily killing the cells [5,6]. These strategies should not be treated as interchangeable because senescence induction may suppress tumour growth but could also permit the persistence of SASP-producing cells, whereas inappropriate systemic suppression of senescence may interfere with beneficial therapy-induced tumour-cell arrest [5,6].

Persistent senescent tumour or stromal cells can secrete a SASP containing cytokines, chemokines, growth factors, proteases, extracellular vesicles, and other mediators. In selected experimental settings, a persistent SASP may remodel the tumour microenvironment, support inflammatory or immunosuppressive signalling, promote CSC-like phenotypes, and contribute to treatment resistance or relapse. These effects are context-dependent and should not be interpreted to mean that all senescent cells are tumour-promoting or that all CSC-like cells arise from senescence [5,6,7].

### 1.3. Why CSCs and Senescence Should Be Considered Together

CSCs and cellular senescence represent biologically distinct but potentially interacting dimensions of treatment failure. Stemness-associated and reversible drug-tolerant states may enable tumour cells to survive therapeutic stress, whereas persistent TIS and the SASP may modify the surrounding microenvironment in ways that support cell survival, immune evasion or acquisition of stemness-associated characteristics. Conversely, senescence may also restrict tumour growth and facilitate immune-mediated clearance. Its consequences therefore depend on the affected cell type, inducing treatment, duration of arrest, SASP composition and immune context [4,7].

Importantly, the available evidence does not yet establish a universal or direct CSC–senescence axis. Most studies examine CSC-associated phenotypes and senescence-related processes separately, and shared modulation of pathways such as Wnt/β-catenin, STAT3, NF-κB or oxidative-stress signalling does not by itself demonstrate functional crosstalk. Direct evidence would require the same compound, within the same cancer or tumour-microenvironment model, to modify a well-defined senescence or SASP and a validated CSC function, followed by an effect on treatment resistance, residual disease or recurrence. Whether plant-derived bioactive compounds can produce such coordinated effects remains an important unresolved question addressed in this review.

### 1.4. Plant-Derived Bioactive Compounds as Multitarget, Adjunctive Candidates

Plant-derived bioactive compounds, including curcumin, resveratrol, epigallocatechin gallate (EGCG), berberine, piperine, and shikonin, have attracted interest because they may affect multiple pathways relevant to cancer biology. The term plant-derived bioactive compound is used here rather than dietary phytochemical because some compounds, particularly berberine and shikonin, are primarily investigated as medicinal, supplemental or purified pharmacological agents rather than through conventional dietary exposure. In preclinical models, these compounds have been associated with modulation of Wnt/β-catenin, Hedgehog, Notch, PI3K/Akt/mTOR, redox, inflammatory, and stress-response signalling. Some studies also reported changes in CSC-associated markers or functions, senescence- or SASP-related processes, and responses to chemotherapy or radiotherapy [8,9].

However, multitarget activity should not be equated with simultaneous modulation of CSCs and senescence or with established clinical efficacy. Evidence varies considerably across compounds, cancer types, model systems, formulations, doses, and experimental endpoints. Furthermore, many active concentrations reported *in vitro* may not be achievable as unbound systemic exposures in humans. Poor bioavailability, variable pharmacokinetics, potential-specific drug interactions, tumour heterogeneity, and limited clinical evidence remain major barriers to translation. Plant-derived bioactive compounds should therefore be considered as investigational adjunctive candidates for modulating recurrence- and resistance-associated mechanisms rather than clinically established alternatives to standard treatment [8,9].

### 1.5. Scope and Organisation of This Review

This critical narrative review evaluates preclinical and translational evidence concerning plant-derived bioactive compounds as modulators of CSCs and cellular senescence in relation to treatment resistance and recurrence-associated mechanisms. Curcumin, resveratrol, EGCG, berberine, piperine and shikonin were selected because published studies report effects on CSC-associated phenotypes, senescence or SASP-associated processes, treatment sensitisation, or combinations of these outcomes. Their inclusion does not imply that every compound has been shown to regulate both biological axes within the same experimental model.

This review examines whether findings from these two largely parallel bodies of evidence support an integrated CSC–senescence framework. The evidence is classified as direct cross-axis evidence, intermediate mechanistic evidence, or marker- and pathway-level support. Modulation of shared pathways is considered indirect evidence unless a causal relationship between a defined senescence or SASP and a validated CSC function has been demonstrated within the same experimental model.

Section 2 discusses CSC-associated markers, functional assays, and signalling pathways. Section 3 outlines cellular senescence, the SASP, and TIS, while Section 4 critically evaluates the evidence for CSC–senescence crosstalk. Section 5 and Section 6 assess the effects of selected plant-derived bioactive compounds on CSC-associated and senescence-related phenotypes, respectively. Section 7 considers their preclinical therapeutic implications, and the subsequent sections examine human evidence and translation limitations, including exposure, formulation, safety and drug interactions. The review aims to determine where evidence is direct, where it remains mechanistically suggestive, and which experimental requirements must be met before claims concerning resistance or recurrence can be justified [2,3,4,5,6,7,8,9].

## 2. Methodology

A comprehensive literature search was conducted using the MEDLINE/PubMed database to identify studies examining the effects of dietary phytochemicals on cancer stemness, cellular senescence, cancer recurrence, and therapeutic resistance. Articles published between June 2000 and July 2026 were considered. Search terms were used individually and in combination, including “dietary phytochemicals,” “bioactive compounds,” “natural compounds,” “cancer stem cells,” “cancer stemness,” “cellular senescence,” “therapy-induced senescence,” “senolytics,” “cancer recurrence,” “tumour relapse,” “drug resistance,” and “chemoresistance.” Specific phytochemical classes and compounds, including polyphenols, flavonoids, carotenoids, alkaloids, terpenoids, curcumin, resveratrol, epigallocatechin gallate, quercetin, sulforaphane, and genistein, were also incorporated into the search strategy.

Original *in vitro*, *in vivo*, and human studies were included if they investigated the effects of plant-derived bioactive compounds on cancer stem-cell characteristics, senescence-associated pathways, tumour recurrence, or anticancer drug resistance. Eligible studies examined relevant outcomes such as self-renewal, sphere formation, stemness-marker expression, epithelial–mesenchymal transition, senescence-associated secretory phenotype, cell-cycle arrest, apoptosis, tumour initiation, metastasis, treatment response, or recurrence. Review articles were used to identify additional relevant primary studies but were not included as primary evidence. Studies unrelated to cancer, plant-derived bioactive compounds, cancer stemness, or cellular senescence were excluded, as were articles lacking sufficient mechanistic or experimental information.

The titles and abstracts of retrieved records were screened for relevance, followed by full-text assessment of potentially eligible articles. The included studies were narratively synthesised according to phytochemical class, cancer type, experimental model, molecular target, effects on cancer stemness or cellular senescence, and potential roles in preventing recurrence and overcoming drug resistance. Evidence from cell-based studies, animal models, and human investigations was clearly distinguished to avoid overstating clinical applicability. The major characteristics, mechanisms, and findings of the included studies are summarised in the corresponding tables and figures.

## 3. Cancer Stem Cells: Identification, Markers, and Signalling Pathways

Cancer stemness is a functional and potentially reversible cellular state that is central to understanding tumour heterogeneity, therapeutic adaptation and minimal residual disease. Rather than representing a uniform and permanently fixed cell population, CSC-associated states can emerge dynamically through genetic, epigenetic, metabolic, and microenvironmental influences. This plasticity complicates both CSC identification and therapeutic targeting, because the expression and functional relevance of putative CSC markers differ among cancer types and treatment settings [10,11]. A rigorous evaluation of CSCs therefore requires integration of cancer-appropriate marker combinations, functional assays, and pathway analysis. This section summarises the principles used to identify CSC-associated populations, the major markers and signalling pathways involved, and the limitations that should be considered when interpreting CSC-directed evidence.

### 3.1. What Are Cancer Stem Cells?

Cancer stem cells are a functional and plastic subpopulation of tumour cells that can sustain tumour growth, regenerate tumours, and contribute to intratumoral heterogeneity under appropriate experimental and clinical conditions [12,13]. Their defining properties include self-renewal, tumour-initiation capacity, differentiation potential, and adaptation to therapeutic or microenvironmental stress. Cancer-stem-cell-associated states are therefore linked to metastasis, treatment resistance, residual disease, and recurrence; however, they should not be regarded as a fixed population or as the apex of a hierarchy in every tumour [14,15].

### 3.2. Cancer Stem-Cell Identification: Marker Combinations and Functional Assays

No single molecular marker is sufficiently specific to identify CSCs across cancer types. CSC identification should therefore combine cancer-appropriate marker panels with functional evidence. Commonly used markers include CD44, CD133, ALDH1, EpCAM, LGR5, and the pluripotency-associated transcription factors NANOG, OCT4, and SOX2; their interpretation depends on the tumour type, cellular state, and assay used (Table 1) [16,17]. Functional assays provide the evidentiary standard that marker expression alone cannot provide. Tumoursphere assays assess survival and self-renewal under non-adherent, serum-free conditions, whereas clonogenic assays evaluate the colony-forming capacity of individual cells. However, sphere formation alone does not establish selective CSC targeting because changes in sphere number or size may also reflect general effects on cell survival or proliferation. Stronger evidence requires viability-adjusted assays, serial sphere-formation or clonogenic assessment, limiting-dilution analysis and, where feasible, *in vivo* tumour-initiation experiments. Differentiation assays and therapy-resistance experiments can further characterise CSC-associated phenotypes but should be interpreted together with marker combinations and functional data rather than as stand-alone evidence of CSC identity or selective targeting [17].

The following markers are representative rather than universal. In Section 5, evidence for a phytochemical should be considered strongest when it reduces an appropriate marker combination together with a functional CSC endpoint, such as sphere formation, clonogenicity, or tumour initiation.

#### Representative Cancer-Stem-Cell-Associated Markers

CD44 is a broadly expressed hyaluronan receptor and is frequently used in combination with markers such as CD24 or ALDH1. In selected breast, ovarian, and pancreatic cancer models, CD44-positive populations have been associated with self-renewal, EMT, invasion, and therapy resistance. Hyaluronan–CD44 signalling can activate PI3K/AKT and, in specific models, NANOG/STAT3-associated MDR1 expression; nevertheless, CD44 is not CSC-specific, and its isoforms and biological significance vary by cancer type [18,19].

CD133 (PROM1) is a commonly used CSC-associated surface marker in glioblastoma and several epithelial malignancies. CD133 expression has been associated with clonogenicity, tumour initiation, and therapy resistance, but it should not be assumed to regulate PI3K/AKT or MAPK identically in every cancer. Its interpretation is limited by expression in non-CSC populations and by antibody- and glycosylation-dependent detection [20,21,22].

ALDH activity is a functional readout related to aldehyde detoxification, redox control, and retinoic-acid metabolism. ALDH1A1 and ALDH1A3 are frequently implicated in CSC-associated phenotypes, although the dominant isoform and mechanism vary between tumour types. ALDH activity should therefore be reported with the relevant isoform, cancer model, and companion marker or functional assay [23,24,25].

EpCAM (CD326) is an epithelial cell-surface protein that can be associated with CSC-like phenotypes in selected epithelial cancers. Its regulated intramembrane proteolysis releases EpICD, which can participate in transcriptional complexes linked to proliferation and Wnt-associated gene expression. However, EpCAM is heterogeneous and may decline during EMT; it should not be used as a stand-alone CSC marker [26,27].

LGR5 (also known as GPR49) is a stem-cell-associated receptor that potentiates Wnt signalling through R-spondin-mediated neutralisation of RNF43 and ZNRF3, thereby increasing Frizzled/LRP5/6 receptor availability. In colorectal and other cancer models, LGR5-positive and LGR5-negative tumour-cell populations can show reversible plasticity. Because LGR5 is also expressed in normal adult stem cells and CSC populations may exist without LGR5, it should be interpreted with functional and tumour-specific evidence [28,29,30].

NANOG, OCT4 (POU5F1), and SOX2 form a pluripotency-associated transcriptional network and may support self-renewal, EMT, metabolic adaptation, and therapy resistance in selected cancer models. Their expression is context dependent and also occurs in normal developmental or regenerative programmes; co-expression with other CSC markers and functional validation are therefore required [31,32].

### 3.3. Core Signalling Pathways Regulating Cancer Stem-Cell Phenotypes

Wnt/β-catenin, Notch, and Hedgehog are conserved developmental pathways that can regulate self-renewal, plasticity, survival, and differentiation. Their dysregulation may promote CSC-associated phenotypes, but therapeutic inhibition is complicated by their essential roles in normal tissue maintenance [33,34].

PI3K/Akt/mTOR, NF-κB and JAK/STAT3 signalling contribute to survival, inflammatory signalling, cellular plasticity and treatment resistance in selected CSC models [13]. However, modulation of these pathways alone does not establish a CSC-specific effect. In this review, pathway modulation is therefore classified as indirect mechanistic evidence unless accompanied by functional assessment of self-renewal or tumour-initiating capacity, using appropriately controlled serial sphere-formation assays and, where feasible, limiting-dilution tumour-initiation or serial transplantation experiments [4,17,20]. Clonogenicity can provide complementary evidence but does not independently establish cancer stemness.

**Table 1:** Cancer-stem-cell-associated markers and their context-dependent limitations.

CSC-Associated Markers	Representative Cancer Contexts	Main Interpretive Role	Recommended Companion Evidence	Key Limitation	References
**CD44**	Breast, ovarian, pancreatic	CSC-associated adhesion receptor; linked to PI3K/AKT signalling and EMT-associated states	CD24, ALDH1 co-analysis; Limiting-dilution tumour initiation, serial transplantation, validated sphere assays	Not CSC-specific; also expressed on non-stem tumour and normal cells. Best established in breast cancer	[17,18,23]
**CD133**	Glioblastoma, pancreatic, lung, prostate	CSC-associated markers linked to stem-like enrichment and clonogenicity	ALDH1/CD44 co-analysis; clonogenic, sphere and limiting dilution assays; serial transplantation	Detection depends on glycosylation/antibody clone; CD133 cells can also initiate tumours	[17,22,23]
**ALDH1A1/ALDH1A3**	Breast, ovarian, colorectal, lung	CSC-associated aldehyde detoxification and redox regulation	ALDH activity plus isoform-specific expression CD44/CD24; clonogenic and limiting-dilution assays treatment-response data	ALDRFLUOR does not resolve isoforms; role is tumour-dependent	[17,18,24]
**EpCAM**	Colorectal, breast, lung, hepatocellular	Epithelial-state-associated marker; EpICD/Wnt signalling link	CD44/CD133 co-analysis; EpICD/Wnt readouts; sphere assay and tumour initiation	Heterogeneous; may decrease during EMT	[17,27]
**LGR5**	Colorectal, gastric, ovarian, hepatocellular	CSC-associated R-spondin-Wnt receptor; linked to plasticity	Wnt target genes; organoid formation, tumour initiation, lineage tracing	Also expressed in normal stem cells; commonly observed in colorectal models	[17,28]
**NANOG/OCT4/SOX2**	Breast, colorectal, gastric, liver, brain	CSC-associated pluripotency-related transcriptional state	Co-expression panels with nuclear localisation; sphere, differentiation and tumour-initiation assays	Context-dependent and does not show self-renewal	[17,31]

In the absence of Wnt ligand, the β-catenin destruction complex—comprising APC, Axin, CK1α, and GSK3β—phosphorylates β-catenin. β-TrCP, as part of an SCF E3 ligase complex, recognises phosphorylated β-catenin and promotes its ubiquitination and degradation. Wnt binding to Frizzled and LRP5/6 activates Dishevelled, reduces destruction-complex activity, and permits β-catenin accumulation and TCF/LEF-dependent transcription of genes relevant to proliferation and stemness. Aberrant Wnt activation can support CSC-associated phenotypes in specific tumour models, but the pathway should be interpreted together with functional endpoints [35,36].

Canonical Notch signalling is initiated by contact between Notch receptors and Delta-like or Jagged ligands on neighbouring cells. Sequential ADAM10/17 and γ-secretase cleavage releases the Notch intracellular domain (NICD), which forms a transcriptional complex with CSL/RBPJ and co-activators to induce HES and HEY family genes. Aberrant Notch activation may support self-renewal, EMT, and therapy resistance and can show crosstalk with Wnt/β-catenin and Hedgehog signalling; normal stem-cell toxicity remains an important therapeutic limitation [37,38].

In the absence of Sonic Hedgehog (Shh), Patched (PTCH) restrains Smoothened (SMO), limiting GLI transcription-factor activation. Shh binding relieves this restraint, allowing SMO-dependent GLI activation and transcription of genes associated with proliferation, survival, EMT, and stemness. Hedgehog dysregulation can support CSC-associated traits in selected cancers, but inhibition may also disturb normal stem-cell function and tissue homeostasis [39,40,41].

Importantly, modulation of pathways shared by CSCs and cellular senescence does not by itself demonstrate causal CSC–senescence crosstalk [6,7,13]. In this review, such pathway changes are classified as indirect mechanistic evidence. Direct cross-axis evidence requires validated stemness functions and clearly defined senescence or senescence-associated SASP endpoints to be examined within the same experimental model [6,7,20]. A causal intervention linking the two processes further strengthens this evidence.

### 3.4. Conclusion

CSC evaluation requires integration of cancer-appropriate marker combinations, functional stemness assays, and signalling-pathway analysis. Marker expression alone is insufficient to establish a CSC population. Based on this framework, Section 5 will evaluate whether plant-derived bioactive compounds reduce CSC-associated markers together with direct functional endpoints, including self-renewal, clonogenicity, and tumour initiation, and whether they suppress Wnt/β-catenin, Notch, Hedgehog, or related stemness pathways.

## 4. Cellular Senescence in Cancer

Cellular senescence is increasingly recognised as a context-dependent component of cancer biology and cancer therapy. Although senescence can suppress tumour development by limiting the proliferation of damaged or oncogene-activated cells, its consequences depend on the cell type, inducing stimulus, duration of persistence, and surrounding tumour microenvironment. In particular, chemotherapy, radiotherapy, and targeted therapies may induce TIS in tumour and non-malignant stromal cells. When senescent cells are efficiently cleared, this response may support tumour control; however, persistent senescent cells and their SASP may, in selected models, promote chronic inflammation, microenvironmental remodelling, therapeutic resistance, and cancer progression [42,43,44]. This section outlines the defining features, molecular pathways, SASP, and context-dependent roles of senescence in cancer.

### 4.1. What Is Cellular Senescence?

Cellular senescence is a state of stable cell-cycle arrest in which cells remain metabolically active but no longer proliferate in response to mitogenic signals [42]. It can be triggered by telomere shortening, DNA damage, oxidative stress, oncogenic signalling, chronic inflammation, and epigenetic alterations [42,43,44]. Although senescence is generally characterised by stable cell cycle arrest, the stability of this state varies with cell type and inducing stimulus. Under certain experimental conditions, senescence-like cells may regain proliferative capacity, and this is known as senescence escape.

Senescence escape describes the re-entry of senescent or senescence-like cells into active proliferation following disruption of the mechanisms maintaining cell-cycle arrest. Genomic instability acquired during senescence can contribute to this process, with the resulting escaped cells gaining aggressive biological cellular traits, such as EMT, malignant transformation, and chemoresistance [45,46].

Senescence escape should be distinguished from genuine reversal of senescence. Senescence escape refers to the re-entry of senescent or senescence-like cells into the cell cycle after disruption of the mechanisms maintaining growth arrest. By contrast, genuine reversal would require restoration of the original presenescent cellular state, which has not been conclusively demonstrated. For example, Beauséjour et al. showed that inhibition of p53 signalling enabled some senescent human cells with low p16 expression to resume proliferation; however, this finding is more cautiously interpreted as escape from an incompletely maintained senescence state rather than complete reversal to the original presenescent phenotype [47].

In cancer, senescence can act as a tumour suppressive barrier by limiting proliferation of damaged or potentially tumorigenic cells [48]. Senescent cells are commonly characterised by stable growth arrest and a combination of markers, including p53/p21 or p16/Rb pathway activation, SA-β-gal activity, altered morphology, and, in some contexts, SAHF and SASP expression [49,50,51]. SA-β-gal activity, p16INK4a and p21 are commonly used indicators of senescence, but none is individually sufficient to establish a senescent phenotype. Morphologically, senescent cells commonly show enlarged, flattened, and irregular shapes [51]. In normal tissues, transient senescence can contribute to repair; however, persistent accumulation may promote ageing, chronic inflammation, and impaired tissue regeneration [52].

### 4.2. Molecular Pathways Regulating Senescence

Cellular senescence is induced and maintained through interconnected tumour suppressor pathways, major pathways include p53/p21 and p16/Rb1. However, this can vary according to the cell type, inducing stimulus, and genetic context. Other molecular pathways such as cGAS-STING and mechanistic target of rapamycin (mTOR) can also induce senescence [53,54].

DNA-damaging stressors and oxidative stress activate the DNA-damage response (DDR). ATM and ATR signalling stabilises p53, in part through regulation of MDM2, and activated p53 induces the cyclin-dependent kinase inhibitor p21, contributing to cell-cycle arrest [55].

Persistent DDR signalling may also induce p16INK4a (encoded by CDKN2A). p16INK4a inhibits CDK4/6, thereby maintaining Rb in a hypophosphorylated state. Hypophosphorylated Rb restrains E2F-dependent transcription and limits S-phase entry, contributing to stable cell-cycle arrest [56,57,58,59].

### 4.3. Senescence-Associated Secretory Phenotype

The SASP is a bioactive secretome produced by many, but not all, senescent cells. Its composition varies with cell type, senescence inducer, and tissue context and may include cytokines (e.g., IL-6, IL-8, and TNF-α), chemokines, growth factors (e.g., TGF-β and VEGF), DAMPs, matrix remodelling enzymes, and extracellular vesicles [60,61,62]. Accordingly, SASP can exert tumour suppressive or tumour promoting effects. In an acute and efficiently resolved setting, SASP can reinforce growth arrest, recruit immune cells, and support clearance of damaged oncogene- or therapy-induced senescent cells. These effects may help limit the proliferation of premalignant or damaged tumour cells [62].

When immune clearance is inefficient, persistent SASP may sustain chronic inflammation, angiogenesis, and tumour-microenvironment remodelling [42,63]. Senescent cancer-associated fibroblasts (senCAFs), for example, can promote breast cancer progression by producing extracellular-matrix components that impair natural killer cell infiltration and cytotoxicity [64]. Reduced immune surveillance may thereby favour tumour cell survival and growth. In specific experimental models, IL-6 and IL-8 have been associated with tumour cell proliferation, migration, invasion, and EMT-related programmes [65,66,67]. These changes may support CSC-associated phenotypes and therapeutic resistance [68]. TNF-α can also sustain inflammatory NF-κB- and STAT-related signalling, oxidative stress, and DNA-damage responses [69,70]. Its effects are context-dependent.

SASP-associated chemokines, including CXCL1, CXCL2, CXCL8/IL-8, and CXCL12, can influence immune cell recruitment and tumour cell behaviour through G protein-coupled receptor signalling. In cancer models, these mediators have been associated with angiogenesis, proliferation, migration, and EMT [61,62,63,64,71,72,73,74]. However, their effects depend on the cellular source and tumour context. TGF-β is produced by tumour, stromal, and immune cells and can contribute to senescence, fibrosis, stromal remodelling, and immune regulation in a context-dependent manner [75,76]. Other SASP-associated factors, including VEGF, DAMPs, MMPs, and extracellular vesicles, may alter vascular permeability, inflammatory signalling, extracellular matrix remodelling, and intercellular communication [63,77,78,79]. Their specific contribution to cancer-associated senescence should be interpreted according to the experimental model.

### 4.4. Therapy-Induced Senescence

Cancer therapies, including chemotherapy, radiotherapy, and targeted therapy, can induce TIS through various mechanisms such as DNA damage, oxidative stress, and mitochondrial dysfunction [80,81]. Targeted agents such as CDK4/6 inhibitors can induce senescence through sustained cell-cycle arrest [82]. TIS most commonly activates p53/p21- and p16/Rb-associated growth-arrest programmes and can be accompanied by SASP secretion, although the pathways involved vary according to treatment, cell type, and genetic context. Additional signalling pathways, such as cGAS-STING and mTOR, can contribute to the regulation of senescence-associated inflammatory and secretory responses [54,83]. TIS has a dual role: acute senescence may suppress tumour growth and facilitate immune clearance, whereas persistent senescent tumour or stromal cells may generate a microenvironment that favours tumour progression and, in selected models, senescence escape.

TIS can occur in both tumour cells and surrounding stromal cells within the TME, as demonstrated in chemotherapy-treated tumour tissues and primary stromal fibroblasts [84]. The immune system regulates senescent cells by immune surveillance. During the early tumour suppressive phase of TIS, senescent tumour cells release SASP factors that recruit natural killer (NK) cells, macrophages and CD4^+^/CD8^+^ T cells. In a mouse liver carcinoma model, restoration of p53 induced tumour-cell senescence and promoted NK-cell recruitment, while NKG2D deficiency or antibody-mediated NKG2D blockade delayed elimination of the senescent tumours. These findings demonstrate that NKG2D-dependent NK-cell surveillance is one of the mechanisms contributing to the immune-mediated clearance of senescent tumour cells, therefore enhancing tumour suppression [85].

Persistent TIS can become tumour-promoting when senescent cells accumulate and are not efficiently cleared. Age-related immune dysfunction may reduce clearance of senescent cells [86]. Persistent SASP may then contribute to an immunosuppressive tumour microenvironment, impair antitumour immunity, and support tumour-cell survival. These context-dependent effects have been associated with treatment resistance and cancer relapse [87,88].

Not all therapy-induced senescent tumour cells remain stably arrested. Emerging evidence indicates that a subset can escape senescence and re-enter the cell cycle [87,88]. Depending on the model, therapy-induced senescent cancer cells may upregulate immune checkpoint ligands, including PD-L1 and PD-L2, which can impair T-cell-mediated clearance and promote persistence of senescent cells within the tumour microenvironment [87,89]. Escaped cells may generate more aggressive proliferative clones with increased invasiveness and resistance and could thereby contribute to relapse [90].

Persistent senescence can become protumorigenic when senescent tumour or stromal cells are not efficiently cleared. Senescent CAFs can remodel the tumour microenvironment through SASP-associated signalling and thereby promote tumour-cell proliferation, invasion, and metastatic dissemination [64]. In specific experimental models, SASP mediators such as IL-6, IL-8, and TGF-β have been associated with STAT3-, NF-κB-, and EMT-related programmes in neighbouring tumour cells [56,65,66,67]. These programmes may enhance stemness-associated phenotypes, therapeutic resistance, and recurrence potential [69].

However, activation of shared pathways such as STAT3, NF-κB and EMT-related signalling does not by itself demonstrate causal crosstalk between cellular senescence and CSCs [13,65,69]. In this review, such pathway changes are classified as indirect mechanistic evidence. Direct cross-axis evidence requires a defined senescence or SASP and validated functional stemness endpoints to be examined within the same experimental model, preferably using a causal intervention that links the two processes.

## 5. Crosstalk Between Cancer Stemness and Cellular Senescence

**Figure 1:**
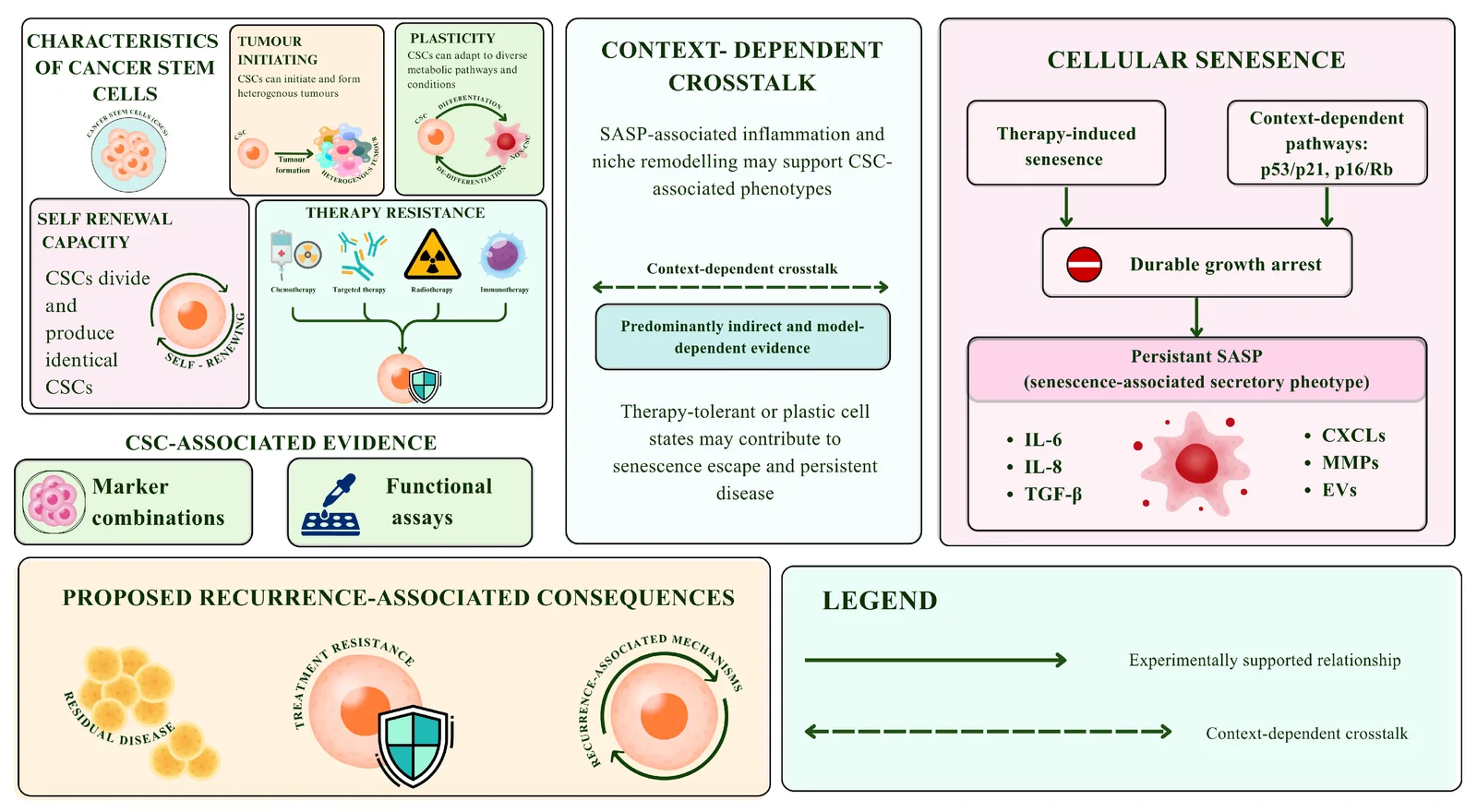
Context dependent crosstalk between CSC-associated states and cellular senescence. CSC-associated cells exhibit self-renewal, plasticity and therapy resistance, while senescent cells may release inflammatory and remodelling factors through the senescence-associated SASP. Their proposed crosstalk may contribute to residual disease and recurrence, but evidence remains largely indirect and model-dependent. Solid arrows indicate experimentally supported relationships; the dashed arrow indicates context-dependent crosstalk. Created in Canva. Naseer, S. (2026). https://canva.link/ona3b9v8z2cochr.

CSCs and cellular senescence should be considered as dynamically connected, context-dependent processes in cancer. Therapy-induced senescence and persistent SASP may influence the tumour microenvironment and, in selected models, support CSC-like phenotypes, therapeutic adaptation, and recurrence-related mechanisms. Conversely, CSC plasticity may be associated with senescence escape and persistent disease. The conceptual framework of this bidirectional and context-dependent relationship is summarised in Figure 1.

### 5.1. Conceptual Framework: The Duality of Senescence

Cellular senescence and stemness are not uniformly opposing states. Stable tumourcell senescence can act as an antiproliferative response. However, when senescent tumour or stromal cells persist after therapy or are not efficiently cleared, their secretory programme can remodel the tumour microenvironment. In specific experimental models, SASP mediators such as IL-6, IL-8, and TGF-β can activate JAK/STAT3-, NF-κB-, and EMT-associated programmes in neighbouring tumour cells, thereby promoting stemness-associated phenotypes, therapeutic resistance, and recurrence potential. The magnitude and direction of these effects depend on cell type, senescence inducer, tumour type, and immune context [65,91].

The central premise of this section is therefore not that every senescent cell generates CSCs. Rather, persistent senescence and harmful SASP signalling may create conditions that select for, maintain, or induce CSC-like phenotypes. Conversely, pre-existing CSCs may survive treatment, while CSC-like properties may be selected or acquired during senescence escape. This relationship is bidirectional and context-dependent.

### 5.2. SASP-Mediated Induction of CSC Phenotypes

Evidence concerning CSC senescence crosstalk was classified into three levels for this review. Direct cross-axis evidence refers to studies in which cellular senescence or the SASP was verified and causally linked to a validated functional stemness outcome within the same experimental model, as exemplified by senescence-associated reprogramming studies [91]. Intermediate mechanistic evidence includes studies in which a senescence-associated mediator or pathway was causally linked to a functional stemness outcome, but senescence and stemness were not directly examined together within the same model, or in which both were examined but a causal contribution of senescence was not established. Studies examining SASP-associated cytokines and functional stemness illustrate the importance of this distinction [65]. Marker/pathway only support refers to studies reporting changes in CSC-associated markers or shared signalling pathways without validated functional stemness assays or direct causal testing of the senescence–stemness relationship. This distinction reflects the need to separate marker expression from functional tumour-initiating capacity [12,20]. SASP composition is heterogeneous; no single molecule should be described as the dominant SASP factor in every model. IL-6 is one commonly studied SASP cytokine and can contribute to JAK/STAT3 activation, whereas IL-8/CXCR1/2 and TGF-β signalling can support EMT, motility, and stemness-associated phenotypes in selected models. Extracellular matrix remodelling enzymes, including MMPs, may further modify the microenvironment, but their effects should not be inferred to be uniformly stemness-promoting [65,92].

Direct evidence for the senescence–stemness link should be distinguished from broader inflammatory microenvironment evidence. Cahu et al. provided direct cross-axis evidence that SASP-derived chemokines (RANTES, IP-10) can favour the emergence of cancer stem-like cells in a myeloma model, using conditioned medium transfer and CHK2 knockdown to establish causality [92]. The study by Ortiz-Montero et al. similarly reported direct cross-axis evidence: senescence conditioned medium induced functional stemness in MCF-7 cells, measured as mammosphere self-renewal across serial passages and multilineage differentiation. Neutralising IL-6/IL-8 antibodies allows reversal of these effects, and recombinant IL-6/IL-8 alone can reproduce them, supporting the capacity of senescence-associated IL-6 and IL-8 to reinforce inflammatory and tumour-promoting signalling in breast cancer cells [65]. Both studies show direct cross-axis evidence, with both demonstrating functional CSC outcomes causally linked to senescence/SASP signalling within the same model, in contrast to the following studies, which support only intermediate or marker/pathway-level associations.

Additional studies provide intermediate mechanistic evidence but do not directly demonstrate CSC senescence crosstalk. In a breast cancer model, tumour-associated macrophage-derived IL-6 increased ALDH1 activity, Sca-1 expression, mammosphere formation and the expression of stemness-associated transcription factors. Neutralisation of IL-6 or pharmacological inhibition of STAT3 attenuated these effects [93]. Because the IL-6 was derived from tumour-associated macrophages rather than senescent cells, this study does not provide direct evidence of SASP-mediated CSC induction. Nevertheless, its causal interventions and functional mammosphere assay support its classification as intermediate mechanistic evidence for an IL-6/STAT3 stemness pathway that may also operate downstream of SASP.

Similarly, recombinant IL-8 promoted CSC-associated features in H446 small cell lung cancer cells, including tumoursphere formation, whereas IL-8 silencing attenuated this effect [94]. This study provides intermediate mechanistic evidence linking IL-8 to a functional stemness outcome, but it does not establish senescence CSC crosstalk because senescence and SASP were not assessed. Continuous TGF-β exposure also promoted an intermediate EMT state associated with stemness-related phenotypes in breast and skin cancer models [95]. This finding provides intermediate mechanistic support for TGF-β as a shared mediator, although the absence of a defined senescence context and limited causal validation weaken its relevance to direct CSC–senescence crosstalk.

### 5.3. Therapy-Induced Senescence, Reprogramming, and Senescence Escape

Milanovic et al. provided a landmark demonstration that TIS can be associated with stemness reprogramming in defined haematological and epithelial cancer models, *in vivo* functional validation, *in vivo* tumour initiation, and correlative human biopsy data [91]. Notably, this reprogramming was shown to be largely SASP-independent, driven instead by cell intrinsic chromatin remodelling and canonical Wnt signalling—distinguishing this mechanism from the SASP-mediated crosstalk described in Section 5.2 [91]. In Eμ-Myc B-cell lymphoma cells exposed to adriamycin, senescence was accompanied by an adult tissue stem cell signature, increased ALDH and ABC-transporter activity, and enrichment of stemness-associated features. The authors also used T-ALL models to examine a more clearly defined non-self-renewing population. Following therapy-induced senescence and subsequent escape from arrest, cells showed increased colony formation and tumour-initiation capacity in recipient mice [91]. The study meets the direct cross-axis criteria because senescence-incapable controls and Wnt inhibition show that senescence and the Wnt pathway that mediates it are required for the stemness phenotype and are not merely associated with it.

This study should be interpreted carefully. It does not show that all senescent cancer cells become bona fide CSCs. Instead, it supports the conclusion that TIS can create a reprogramming state in which stemness-associated traits emerge or are selected in specific experimental contexts. Persistent epigenetic changes, including altered histone-mark patterns, may help maintain this altered state after cell cycle re-entry; however, the mechanism and generalisability remain model-dependent [91].

Findings in hepatocellular carcinoma are consistent but less conclusive. In HuH7 hepatocellular carcinoma cells, doxorubicin-induced senescence in EpCAM^−^/CD133^−^ non-stem cells was accompanied by increased stemness-gene expression, an expanded EpCAM^+^ fraction and higher tumour incidence in NSG mice (7/10 versus 3/10 injection) [95]. However, the implanted cells were an unsorted Dox-treated population, no senescence-incapable conrol was used, and Wnt was inhibited only chemically, limiting the evidence that senescence was required for the stemness phenotype [96].

### 5.4. Consequences for Therapy Resistance and Recurrence

Persistent senescence and CSC-associated properties may contribute to residual disease, therapy resistance, immune evasion, metastasis, and recurrence.

#### 5.4.1. Evidence Involving CSC Phenotypes

In a myeloma model, genotoxic stress increased a CD138low stem-like population, and senescence-associated secretory signals were implicated in maintaining an undifferentiated state [92]. This result supports a role for the senescent microenvironment in selected models but should not be generalised to all malignancies. Although the study assessed tumour formation in a xenograft model, the proposed SASP/CHK2 mechanism was not causally validated against *in vivo* recurrence or treatment resistance endpoints [92].

Therapy-induced senescent tumour cells can also influence immune surveillance. In pancreatic and mammary tumour models, intratumoral senescent cells expressing PD-L2 limited chemotherapy efficacy; PD-L2 deficiency or blockade enhanced CD8 T-cell-associated tumour control and reduced recruitment of myeloid-derived suppressor cells [89]. This study shows senescent-cell persistence and immune evasion *in vivo* rather than CSC induction. It reaches the level of animal treatment response but contains no CSC readout and is therefore not classified within the CSC-crosstalk evidence hierarchy above. These data indicate that persistent senescent cells may evade immune clearance and thereby remain within the tumour after therapy.

#### 5.4.2. Senescence Persistence Evidence Without CSC Readout

Senescent cells in normal host tissues may also affect recurrence-related outcomes. In murine breast cancer models, elimination of chemotherapy-induced senescent cells in the host reduced metastatic burden and delayed tumour regrowth [97,98]. As with the PD-L2 findings above, these studies establish a senescence-recurrence link without direct assessment of CSC phenotypes, and as such are not classified within the CSC-crosstalk hierarchy. Nevertheless, these findings support the therapeutic rationale for targeting persistent senescent cells, but the magnitude of benefit and the optimal senolytic, senomorphic, or immune-based strategy remain to be determined in human cancer.

Together, these findings occupy different positions on the mechanism to human continuum. The direct cross-axis SASP studies are supported at the level of *in vitro* functional validation, with no *in vivo* treatment or recurrence data [65,92]. Milanovic et al. [91] provide the most extensive evidence in this section, extending from mechanistic experiments to *in vivo* tumour-initiation assays using limiting-dilution transplantation in lymphoma and leukaemia models. They also observed nuclear β-catenin enrichment in relapsed compared with primary murine lymphomas after cyclophosphamide treatment and reported correlative human biopsy data findings. These relapse findings are observational; they do not show that an intervention prevented recurrence. The senescence persistence and clearance studies assessed *in vivo* treatment or recurrence-related outcomes but did not include CSC readout [89,97,98]. No interventional human evidence currently supports senescence-directed or CSC-directed treatment strategies targeting this proposed axis. The evidence levels and limitations of the studies discussed in Section 5.2, Section 5.3 and Section 5.4 are summarised in Table 2.

### 5.5. Conclusion and Transition

Overall, senescence can promote CSC-associated phenotypes in selected experimental models through secreted SASP factors, as demonstrated by Cahu et al. and Ortiz-Montero et al. [65,93], or through cell-intrinsic reprogramming, as reported by Milanovic et al. [91]. However, findings involving TAM-derived IL-6 [94], recombinant IL-8 [95], TGF-β [96], and the hepatocellular carcinoma model investigated by Karabicici et al. [92] require interpretation according to their functional endpoints and the extent of causal validation of the senescence–stemness relationship. Collectively, these findings provide a preclinical rationale for combined strategies targeting CSCs, harmful SASP signalling, and persistent senescent cells [7,91,97]. Section 6 and Section 7 examine whether plant-derived bioactive compounds can influence cancer stemness and cellular senescence, respectively, while Section 8 discusses the therapeutic implications and translational limitations of this approach.

## 6. Plant-Derived Bioactive Compounds Targeting Cancer Stemness

Plant-derived bioactive compounds may influence CSC-related phenotypes through the modulation of multiple, interconnected signalling pathways, including Wnt/β-catenin, Notch, Hedgehog, PI3K/Akt/mTOR, NF-κB, and STAT3. These pathway-associated effects may be reflected in changes in CSC marker combinations and functional endpoints, such as sphere formation, self-renewal, clonogenicity, EMT/invasion, and therapy sensitivity. However, the available evidence remains predominantly preclinical Table 3 and should be interpreted according to the specific compound, cancer model, dose, and functional assay used.

### 6.1. Plant-Derived Bioactive Compounds Targeting Cancer Stemness

CSCs are best regarded as having a functional and potentially reversible cellular state rather than being a universally fixed tumour-cell subpopulation. Cells exhibiting CSC-associated states may demonstrate self-renewal, tumour-initiation capacity and adaptation to therapeutic or microenvironmental stress. However, no single marker is universally specific for these states. CSC-directed effects should therefore be evaluated using cancer-type-appropriate marker combinations together with validated functional assays, such as serial sphere formation, clonogenicity and limiting-dilution tumour-initiation experiments [99,100,101].

This section evaluates the strength of preclinical evidence that selected plant-derived bioactive compounds modulate CSC-associated markers, functional stemness phenotypes or treatment responses in defined cancer models. Molecular changes in Wnt/β-catenin, Hedgehog, PI3K/Akt/mTOR, or related pathways are interpreted as mechanistic support only when they are accompanied by relevant CSC readouts. The evidence remains predominantly preclinical and should therefore not be interpreted as proof of clinical prevention of recurrence or drug resistance [99,100,101]. Sphere formation alone does not establish selective CSC targeting because reduced sphere number or size may reflect general cytotoxicity or impaired proliferation. Stronger evidence combines viability-controlled assays and serial sphere formation with comparison against non-CSC-enriched tumour populations [4,17]. Clonogenic assays provide complementary evidence, while limiting-dilution tumour-initiation and serial transplantation experiments can more directly assess tumour-initiating capacity [20]. Normal-cell controls should additionally be included to assess tumour selectivity.

#### 6.1.1. Curcumin

For this section, evidence was interpreted along a continuum defined for this review. Marker/pathway-level evidence refers to changes in CSC-associated markers or signalling pathways without validated functional assays; marker expression alone does not establish functional cancer stemness [12,100]. Functional CSC-associated evidence includes appropriately controlled serial sphere formation, clonogenicity, differentiation or tumour-initiation assays [17,20]. Advanced preclinical evidence additionally includes *in vivo* validation of tumour initiation, treatment response, residual disease or recurrence [4,20,91,97]. None of these categories should be interpreted as clinical evidence unless supported by appropriately designed human studies.

In breast cancer stem-cell-enriched models, curcumin has been reported to suppress Sonic Hedgehog and Wnt/β-catenin signalling, accompanied by reduced expression of CSC-associated markers (including CD44, CD133, ALDH1A1, OCT4, and NANOG) and decreased mammosphere formation [102,103]. These findings support an inhibitory effect on stemness-associated phenotypes in the reported breast cancer models rather than a general effect on all CSCs. Curcumin also reduced microtentacle formation and invasive/metastatic properties in breast cancer cells with stem-cell-like features [104]. Because BCL-2 is an antiapoptotic protein rather than a CSC marker, changes in BCL-2 should be interpreted as survival-related mechanistic evidence. The limited aqueous solubility and bioavailability of curcumin remain important translational constraints [103].

#### 6.1.2. Shikonin

In chemoresistant non-small-cell lung cancer rat models, shikonin attenuated PI3K/Akt/mTOR signalling and reduced stemness-associated features, including NANOG and OCT4 expression and sphere-forming capacity. Combination treatment with BEZ235 produced an enhanced antistemness effect in the reported model [105]. In lung adenocarcinoma, shikonin has also been associated with reduced malignant phenotypes and Hedgehog-pathway downregulation, when tested with xenograft models [106]. However, GLI1 and GLI2 should be described as Hedgehog-pathway effectors rather than CSC markers. In small-cell lung cancer, shikonin-induced ATF3-mediated ferroptosis provides relevant anticancer context, but this observation does not by itself establish direct CSC targeting. Ferroptosis is an iron-dependent regulated cell-death process and should not be described as apoptosis [107]. Hydrophobicity and poor solubility are factors that lead to the poor bioavailability of shikonin, along with rapid metabolism and clearance when consumed orally [105].

#### 6.1.3. Resveratrol

The CSC-related evidence for resveratrol should be interpreted by cancer type. In pancreatic cancer cells, tested in a KPC mouse model, resveratrol counteracted gemcitabine-induced stemness by targeting SREBP1-dependent lipid metabolism, with reduced sphere-forming ability and enhanced chemosensitivity to gemcitabine [108]. SREBP1 is a metabolic regulator, not a CSC marker. Studies in melanoma or other cancer models provide supportive evidence for antitumour activities of resveratrol, but these findings should not be combined with the pancreatic cancer data as a single universal CSC conclusion [109]. In glioblastoma cells, resveratrol inhibited stem-cell-like properties, EMT-associated migration and invasion, by the effective regulation of the Smad signalling pathway, resulting in suppression of the glioblastoma stem cells, along with EMT [110]. The low bioavailability of resveratrol and uncertainty regarding the clinically achievable exposure remain major translational limitations [109,110].

#### 6.1.4. Epigallocatechin Gallate

EGCG has shown preclinical effects on CSC-associated markers and functional phenotypes in lung and colorectal CSC-like cells through modulation of Wnt/β-catenin signalling. In the reported models, Wnt/β-catenin suppression was accompanied by reduced CSC-associated marker expression and decreased sphere formation [111,112]. In colorectal cancer, EGCG also reduced stem-like-cell phenotypes and enhanced 5-fluorouracil sensitivity, validated through *in vivo* and *in vitro* xenograft model testing [113]. These results support the potential of EGCG to modulate CSC-related pathways and functional readouts in defined preclinical models. Pathway components such as NOTCH1 or BMI1 should be reported as pathway-associated molecules rather than being uniformly classified as CSC markers [111,112,113].

**Table 3:** Preclinical evidence for selected plant-derived bioactive compounds targeting CSC-related phenotypes.

Compound and Reference	Cancer Type and Model	Exposure: Concentration/Dose, Duration, Route and Formulation	CSC Markers and Functional Stemness Assays	Cytotoxicity Control	Molecular Mechanism	Combination and In Vivo Evidence	Resistance or Recurrence Endpoint	Evidence Level and Clinical Evidence	Main Limitation
**Curcumin** [102,103,104]	Breast cancer: *in vivo* and *in vitro* studies done on mouse model and breast cancer cell lines (SUM159, MCF7, MDA-MB231).	*In vitro:* curcumin 0, 10, 20 and 40 µM; curcumin 15, 20 and 30 µM for 24 h.	CD44, ALDH1A1, Nanog, and Oct4 were reduced in a concentration-dependent manner; Inhibition of SUM159 and MCF7 tumoursphere formation in a concentration-dependent manner.	Selective CSC effects were not established by a matched viability-adjusted comparison with non-CSC cells; reduced tumoursphere formation may partly reflect general cytotoxicity.	Suppression of Sonic Hedgehog and Wnt/β-catenin signalling.	Combination of curcumin and mitomycin c, *in vivo* experiment done on mouse model showed reduction of tumour growth.	Reduced chemoresistance	Preclinical evidence: breast cancer cell-line studies and reported animal findings. No clinical evidence that curcumin prevents cancer recurrence or overcomes drug resistance.	Poor water solubility and poor absorption; effective solvent of curcumin is DMSO which is cytotoxic.
**Shikonin** [105,106,107]	NSCLC: docetaxel-resistant A549/D16 and vincristine-resistant A549/V16 cells; A549 lung adenocarcinoma cells and a mouse xenograft model. SCLC: SBC-2 cells and an SBC-2-derived mouse xenograft model.	*In vitro*: shikonin 0, 1 or 2 µM in spheroid assays; shikonin 10, 15 or 20 µM, assessed at 6, 12, 18, 24, 36 and 48 h.	Nanog, OCT4 levels were reduced in a concentration-dependent manner.	SC-specific selectivity versus general cytotoxicity was not established	Specify PI3K/Akt/mTOR; Hedgehog-associated changes; ATF3-mediated ferroptosis.	Shikonin was examined with BEZ235 in resistant NSCLC cells; mouse xenograft tumour-growth findings were reported, without establishing CSC-specific effects *in vivo*.	Drug-resistant A549/D16 and A549/V16 lung cancer cells were tested. Shikonin reduced spheroid formation in these resistant cell models, but treatment-resistance reversal and tumour recurrence were not directly established by the CSC assays.	Preclinical evidence: functional spheroid assays in chemoresistant NSCLC cells, lung adenocarcinoma cell and mouse xenograft studies and SCLC cell and mouse xenograft studies. No clinical evidence of CSC targeting or recurrence prevention was reported.	Poor solubility.
**Resveratrol** [108,109,110]	Melanoma: B16F10, B6 and A375 cells; mouse model of melanoma lung metastasis. Pancreatic cancer: MiaPaCa-2 and PANC-1 cells; KPC mouse model.	Melanoma: specify the 20 and 40 µM cell treatments and 40 mg/kg intraperitoneally in mice; Pancreatic cancer: resveratrol 50 µM with gemcitabine 5 µM for 48 h for the reported sphere assay.	Reduced gemcitabine-associated sphere formation and stemness-marker expression in pancreatic cancer cells: no CSC-specific endpoint reported.	Melanoma cell cytotoxicity was assessed at 20 and 40 µM, but this does not control for selective CSC targeting; interpretation of sphere reduction requires comparison with general effects on cell viability.	SREBP1-dependent lipid metabolism in pancreatic cancer.	Resveratrol reduced lung metastatic tumour growth in mice; resveratrol plus gemcitabine was examined in pancreatic cancer cells; KPC tumour tissue was assessed for stemness-related changes.	Gemcitabine response and gemcitabine-induced stemness-related changes were assessed in pancreatic cancer models. Melanoma lung metastatic growth was assessed. Neither study established prevention of cancer recurrence.	Preclinical cell and mouse studies; no clinical evidence of selective CSC targeting or recurrence prevention in the cited studies.	The melanoma study did not test CSC function. In the pancreatic study, reduced sphere formation and marker expression support CSC-associated effects, but selective CSC targeting and durable prevention of resistance or recurrence remain unproven.
**EGCG** [111,112,113]	Lung cancer: tumoursphere-enriched cells derived from A549 and H1299. Colorectal cancer: DLD-1 and SW480 spheroids; parental and 5-FU-resistant HCT116 and SW480 cells, spheroid-derived CSC-like cells, and a mouse xenograft model using pretreated spheroid-derived cells.	*In vitro*: EGCG 0, 20, 60 or 100 µM for 7 days; *in vitro*: 0, 10, 20, 40 or 60 µM for 6 days;* in vitro* cytotoxicity assays: 25–400 µM for 72 h; xenograft experiment: spheroid-derived cells were treated with EGCG 100 µM for 48 h before implantation.	EGCG reduced CSC-associated marker expression and tumoursphere formation in lung and colorectal cancer cell models; in colorectal spheroid-derived cells, it also reduced CSC-associated properties and enhanced response to 5-FU.	Cell viability and cytotoxicity were assessed over a range of EGCG concentrations in parental and 5-FU-resistant colorectal cancer cells; reduced sphere formation alone does not establish selective CSC targeting, particularly at cytotoxic concentrations.	Wnt/β-catenin suppression was investigated in the lung and colorectal CSC studies.	EGCG enhanced 5-FU effects in resistant colorectal cancer cell models. EGCG-pretreated spheroid-derived colorectal cancer cells showed reduced tumour growth after implantation in a mouse xenograft model.	EGCG increased 5-FU sensitivity in resistant colorectal cancer cell models; tumour recurrence was not assessed.	*In vitro* studies with xenograft support; no clinical evidence of CSC targeting or recurrence prevention.	Effects depend on cancer model and EGCG concentration; high concentrations may cause general cytotoxicity. Xenograft tumour growth does not establish durable CSC depletion or recurrence prevention, and clinically achievable tumour exposure was not demonstrated.
**Berberine** [114,115,116,117]	N2a neuroblastoma cells; SKOV3 and A2780 ovarian cancer cells; murine 4T1 and human MDA-MB-231 breast cancer cells, with a 4T1 tumour-bearing mouse model.	N2a cells: 0, 10, or 20 µg/mL for 72 h. Ovarian cancer cells: 5 µM for 24 h. Breast cancer cell assays: 0, 25, 50, 75, or 100 µM for 48 h; mice: 125 mg/kg/day in 1% sucrose drinking water.	Reduced CD133, β-catenin, N-MYC, SOX2, NOTCH2 and nestin in N2a cells; attenuation of chemotherapy-associated CSC-like characteristics and migration in ovarian cancer models; reduced mammosphere formation and CSC-like cell proliferation in 4T1 and MDA-MB-231 cultures.	Berberine cytotoxicity was assessed by an LDH assay in murine 4T1 and human MDA-MB-231 breast cancer cells across 25–125 µM.	GLI1-associated signalling in ovarian cancer; miR-let-7c and miR-34a-associated changes in breast cancer models.	The ovarian study investigated berberine in the context of chemotherapy-associated CSC-like characteristics. In the 4T1 mouse study, berberine was associated with altered tumour-tissue miRNA expression and lower serum IL-6.	Chemotherapy-associated CSC-like traits and metastasis-related outcomes were examined in the ovarian study. Tumour recurrence and durable reversal of drug resistance were not established by these studies.	Preclinical cell-based evidence with an animal model; no clinical evidence of CSC-targeted efficacy or recurrence prevention.	Marker changes in N2a cells do not establish functional CSC depletion. Functional mammosphere evidence is model-specific, and the mouse findings do not demonstrate that berberine prevents CSC-driven recurrence.
**Piperine** [118,119,120]	HCC: HepG2-derived CD44^+^/CD133^+^ cells. TNBC: MDA-MB-231 cells *in vitro*; EAC solid tumours in mice *in vivo*. Glioblastoma: U87 cells and U87-derived glioblastoma stem-like cells	MDA-MB-231 cells: 100 or 200 µM piperine with doxorubicin; EAC-bearing mice: piperine 50 mg/kg/day intraperitoneally, with doxorubicin 4 mg/kg intraperitoneally once weekly. U87 dose-response assay: 0–500 µM; combination experiments used other piperine/temozolomide concentrations and 24, 48, or 72 h exposures.	Effects were studied in sorted CD44^+^/CD133^+^ HepG2-derived cells. Reduced tumour ALDH-1 staining; this marker alone does not establish reduced CSC function. Survivin and stemness-related readouts were assessed in U87-derived stem-like cells, alongside drug-response experiments.	MDA-MB-231 cell viability was assessed by MTT assay.	PI3K/Akt/mTOR and survivin-associated effects.	Piperine plus doxorubicin improved treatment response in MDA-MB-231 cells and reduced tumour growth in EAC-bearing mice. Piperine plus temozolomide improved response in the U87-derived glioblastoma model *in vitro*.	Enhanced response to doxorubicin and temozolomide was assessed; recurrence was not directly measured.	Preclinical cell and mouse evidence; no clinical efficacy evidence in these studies.	Effects on CSC-associated markers and treatment response do not establish selective CSC elimination or prevention of recurrence. The EAC mouse model is distinct from the MDA-MB-231 human TNBC cell model.

#### 6.1.5. Berberine

Berberine has been reported to attenuate cancer stemness- and EMT-related phenotypes in neuroblastoma cells and to suppress chemotherapy-exacerbated ovarian cancer stem-cell-like characteristics through GLI1-related mechanisms [114]. Additional breast cancer studies suggest that berberine may enhance the response to radiotherapy and may regulate stemness-associated phenotypes through microRNA-related pathways [115,116]. The evidence should be presented separately by model. GLI1, BMI1, HIF-1α, and VEGF are pathway effectors or downstream mediators, rather than CSC markers. Direct functional readouts, including sphere formation, EMT-related phenotypes, or treatment sensitisation, should be prioritised when describing berberine activity [115,116,117].

#### 6.1.6. Piperine

Piperine has shown CSC-related activity in hepatocellular carcinoma, triple-negative breast cancer, and glioblastoma models. In hepatocellular carcinoma, piperine was reported to affect CSC-associated and EMT-related phenotypes [118]. In triple-negative breast cancer, tested *in vivo* and *in vitro* in an animal model (Ehrlich ascites carcinoma solid tumour), piperine enhanced doxorubicin sensitivity and was associated with inhibition of PI3K/Akt/mTOR signalling and CSC-related characteristics [119]. In glioblastoma stem-cell models, piperine-mediated survivin (BIRC5) inhibition reduced colony/sphere formation and increased temozolomide sensitivity [120]. These results should remain separated by cancer type. Piperine may also inhibit drug-metabolising enzymes and transporters, including CYP3A4 and P-glycoprotein. Consequently, apparent chemosensitisation may partly reflect increased intracellular or systemic exposure to the co-administered anticancer drug rather than selective CSC-pathway modulation. Pharmacokinetic analyses and toxicity monitoring are therefore required when evaluating piperine-containing combination treatments [118,119,120].

#### 6.1.7. Summary and Translational Considerations

Collectively, curcumin, shikonin, resveratrol, EGCG, berberine, and piperine have shown the potential to modulate CSC-related markers, pathways, or functional phenotypes in selected preclinical models. The most persuasive evidence includes functional endpoints, such as sphere formation, clonogenicity, treatment sensitisation, or tumour-initiating capacity, rather than marker changes alone [99,100,101,102,106,107,108,109,110,118,119,120].

Nevertheless, the compounds, cancer types, experimental exposures, formulations and CSC-related endpoints differ substantially among studies. Poor bioavailability, limited *in vivo* validation, tumour heterogeneity and potential drug interactions remain important barriers to translation. Moreover, few studies compared CSC-enriched and non-CSC populations under equivalent exposure conditions or assessed residual disease and recurrence directly. These compounds should therefore be regarded as preclinical candidates for modulating CSC-associated mechanisms rather than clinically established strategies for preventing recurrence or overcoming drug resistance [100,101,102,118,119,120].

## 7. Plant-Derived Bioactive Compounds Regulating Cellular Senescence

Cellular senescence is a stress response programme characterised by stable cell cycle arrest and context-dependent changes in cellular function and secretion [6,121]. In cancer, senescence may restrain tumour cell proliferation, whereas persistent senescent cells and their SASP may contribute to inflammatory signalling, treatment-related tissue damage, cancer stemness and relapse [5,7,91,97]. These contrasting effects provide a framework for evaluating plant-derived bioactive compounds according to the target cell population, experimental model, dose, timing and treatment context.

Three mechanistically distinct strategies should be distinguished. Senescence induction aims to establish stable tumour cell arrest; senolysis selectively eliminates established senescent cells; and senomorphic interventions modify the SASP or other senescence-associated features without necessarily killing the cells [6,7]. These strategies have different implications for tumour control and normal tissue protection. Senescence escape, in contrast, involves the resumption of proliferation following disruption of the mechanisms maintaining growth arrest [46,47]. Consequently, reduced senescence-associated markers or renewed proliferation should not automatically be interpreted as beneficial, particularly in tumour cells, where escape may expose enhanced stemness and tumour-propagating potential [91].

The following subsections examine plant-derived bioactive compounds in non-malignant cells and tissues separately from tumour cell models. The reviewed evidence primarily concerns senescence induction or attenuation of senescence-associated phenotypes; none of the studies discussed conclusively demonstrated selective senolytic activity.

### 7.1. Context-Dependent Regulation of Senescence by Plant-Derived Bioactive Compounds

Cellular senescence has context-dependent consequences for cancer. Stable senescence-associated arrest can restrain malignant cell proliferation [5,7,121], whereas persistent senescent cells in normal tissues or the tumour microenvironment may produce a SASP that contributes to tissue dysfunction, immunosuppression, CSC-associated phenotypes and cancer relapses in selected experimental settings [65,97,122]. Accordingly, plant-derived bioactive compounds should not be described as uniformly antisenescent. Three distinct therapeutic strategies should be distinguished: induction of stable senescence in tumour cells, senolytic elimination of established senescent cells, and senomorphic modulation of detrimental SASP or other senescence-associated features without necessarily killing the cells [6,7]. The evidence reviewed in this section primarily concerns senescence induction or attenuation of senescence-associated phenotypes, including effects consistent with senomorphic modulation; convincing selective senolytic activity was not demonstrated. The following studies are evaluated according to their experimental models, implicated pathways, senescence-related endpoints and potential relevance to cancer treatment.

### 7.2. Plant-Derived Bioactive Compounds That Attenuate Detrimental Senescence and SASP

Overall, the reviewed studies highlight the context-dependent role of plant-derived bioactive compounds in regulating cellular senescence. Some compounds may attenuating senescence or cause senomorphic effects in normal cells, such as curcumin, resveratrol, EGCG, berberine, and piperine, while others, such as shikonin, promote senescence in cancer cells.

### 7.3. Senescence Modulation in Non-Malignant Normal Cells and Tissues

#### 7.3.1. MAPK-Related Signalling

In aged mice exposed to normal or high-fat, high-sugar dietary conditions, dietary curcumin was evaluated for its ability to attenuate hepatic cellular senescence and inflammation through MAPK/NF-κB-related signalling [123]. Transcriptomic and primary-hepatocyte analyses indicated modulation of JNK and p38 MAPK pathways. JNK regulation was observed under normal dietary conditions, whereas the reduction in p38 MAPK activation under high-fat, high-sugar conditions was borderline. Thus, the study supports an association between curcumin and reduced age- or metabolic-stress-associated hepatic senescence, rather than a definitive causal conclusion [123].

MAPK activation can contribute to inflammatory NF-κB signalling [123]. In the curcumin-treated groups, reduced p-p65/p65 levels and lower expression of inflammatory genes, including Cxcl2, Cxcl10, and IL-6, were reported, particularly under high-fat, high-sugar dietary conditions. These findings suggest that curcumin may attenuate hepatic senescence-associated inflammation through MAPK/NF-κB modulation. However, the study was conducted in aged non-malignant hepatic models and primarily reflected modulation of senescence-associated inflammatory signalling, limiting direct extrapolation to tumour cell senescence, CSC persistence, recurrence, or treatment resistance [123].

Piperine, a constituent of Piper species including black pepper, was examined in prematurely senescent human diploid fibroblasts (S-HDFs) [124]. The evidence for its senomorphic or senostatic activity therefore derives from non-malignant fibroblasts rather than cancer cells. In this model, piperine was associated with ERK1/2 and JNK regulation, providing a context-specific example of senescence modulation [124].

In premature S-HDFs, piperine was associated with ERK1/2 and JNK phosphorylation, reduced p16INK4a and p21WAF1/CIP1 expression, lower SA-β-gal activity, and decreased secretion of IL-6, IL-8, and TGF-β1 [124]. Piperine treatment also reduced intracellular ROS and increased mitochondrial membrane potential (ΔΨm). The morphology of S-HDFs remained unchanged following treatment [124]. These findings suggest attenuation of senescence-associated phenotypes in this fibroblast model, including mitochondrial function and SASP secretion. The study also reported increased division of senescent S-HDFs following piperine treatment, which the authors interpreted as restoration of senescent cell function [124]. In contrast, experiments in HeLa cancer cells showed that piperine significantly increased IL-6 secretion, demonstrating that its effects on SASP-related signalling differed between senescent fibroblasts and cancer cells [124].

Although the reduction in SA-β-gal activity and SASP secretion was observed, together with restoration of mitochondrial membrane potential (ΔΨm) and reduction of ROS, supporting modulation of the senescence-associated phenotype, increased proliferation of previously senescent S-HDF may also represent cell cycle re-entry or senescence escape rather than a durable and beneficial reversal of senescence. The study did not evaluate long-term proliferative recovery following withdrawal of treatment, tumour cell senescence, CSC persistence, tumour recurrence, or treatment resistance [124].

#### 7.3.2. Resveratrol Modulates Redox Homeostasis Through E2F1/SOD2 Axis

Resveratrol is a natural polyphenol found in grapes, berries, peanuts, and red wine [125]. Its antisenescence activity was investigated in angiotensin-II-induced human vascular smooth muscle cells (VSMCs), a non-cancer model of oxidative-stress-associated senescence [126]. In angiotensin-II-induced VSMCs, resveratrol increased E2F1 and SOD2 expression, reduced ROS, increased SOD activity, and improved mitochondrial membrane potential (ΔΨm). E2F1 silencing weakened the antioxidant effects of resveratrol, whereas E2F1 or SOD2 knockdown diminished its protective effects on oxidative stress and mitochondrial function. Resveratrol was also associated with lower SA-β-gal activity and reduced p21WAF1/CIP1, p53, and γ-H2AX expression; these effects were attenuated by E2F1 silencing [126]. Collectively, the findings support a contributory role of the E2F1/SOD2 axis in resveratrol-mediated protection against VSMC senescence. However, this study was conducted in a non-malignant model and did not evaluate tumour cell senescence, CSC recurrence, tumour recurrence, or treatment resistance.

#### 7.3.3. SIRT1-Linked Protection of Vascular and Cardiac Cells

The SIRT1-linked protection mechanism has been implicated in the antisenescence effects of EGCG and berberine in vascular and cardiac cells. EGCG protects vascular endothelial cells through SIRT1-associated activation of autophagy and inhibition of ferroptosis, whereas berberine protects cardiac cells through the regulation of the Klotho/SIRT1 signalling pathway. The antisenescence effects of EGCG were investigated using both DOX-induced senescence of human umbilical vein endothelial cells (HUVECs) and aged mice, whereas berberine exhibited protective effects in DOX-induced H9c2 cardiomyocyte senescence and aged rats [127,128].

The EGCG study investigated protection of doxorubicin-exposed endothelial cells and vascular ageing rather than direct suppression of cancer-cell senescence [127]. Such normal tissue protection may be beneficial, but its effects should be interpreted cautiously if TIS in tumour cells is also relevant to treatment response.

In doxorubicin-induced senescence of HUVECs, EGCG increased SIRT1-associated signalling. Pharmacological SIRT1 inhibition (EX527) and siRNA-mediated SIRT1 knockdown attenuated the EGCG-associated effects, supporting SIRT1 involvement. EGCG was associated with increased autophagy, reduced ferroptosis-related stress, lower p53, p21WAF1/CIP1, and p16 expression, and reduced SA-β-gal staining [127]. These results support a SIRT1-linked protective mechanism in endothelial cells, not direct evidence for CSC regulation or recurrence prevention.

Berberine demonstrated protective effects against doxorubicin-induced senescence in H9c2 cardiomyocytes through a Klotho/SIRT1-associated pathway. Berberine or exogenous Klotho increased SIRT1-associated signalling and reduced p21WAF1/CIP1, p53, and SA-β-gal positivity, whereas Klotho knockdown abolished these effects and returned the markers towards doxorubicin-only levels [128]. This supports a requirement for Klotho in the antisenescent effect of berberine in this cardiac-cell model. The relevance to cancer-associated senescence, CSCs, recurrence, and resistance remains to be determined.

Collectively, these findings suggest that both EGCG and berberine attenuate senescence, in which the effect of EGCG is exerted via SIRT1-mediated autophagy and ferroptosis regulation, whereas berberine is associated with the Klotho/SIRT1 pathway. However, the senescence-modulating effects of EGCG and berberine were primarily investigated in non-malignant endothelial, cardiac cells, and aged tissue. Therefore, their direct relevance to tumour cell senescence, CSC persistence, tumour recurrence, or treatment resistance remains unclear.

### 7.4. Senescence Modulation in Tumour Cells

Shikonin, a compound derived from *Lithospermum erythrorhizon*, has been investigated for its ability to induce senescence in tumour cells [129]. This differs from the studies discussed above, which primarily examined attenuation of senescence-associated phenotypes in non-malignant tissues.

Zheng et al. studied shikonin in A549 (p53-wild-type) and H1299 (p53-null) lung cancer cells and their corresponding xenograft models [129]. Shikonin increased reactive oxygen species (ROS), DNA-damage markers and p21 expression in both cell lines. In A549 cells, p53 contributed to p21 induction, whereas p21 induction in H1299 cells occurred independently of p53. Silencing p21 reduced the senescence response in both models, supporting its functional involvement [129].

Increased senescence-associated β-galactosidase (SA-β-gal) staining, senescence-associated heterochromatin foci and enlarged, flattened cell morphology further supported senescence induction. Shikonin also reduced EdU incorporation and cyclin D1 expression and increased G0/G1 cell-cycle arrest, consistent with reduced proliferation. In both xenograft models, treatment reduced tumour weight and increased SA-β-gal activity and markers of DNA damage and p21 signalling [129].

These findings support a tumour-cell senescence response, but the study did not establish whether growth arrest persisted after treatment withdrawal or exclude apoptosis as a contributing mechanism. Functional cancer stem cell endpoints, residual disease, recurrence and treatment resistance were also not assessed.

Although Table 4 summarises the experimental concentrations and doses, their translational plausibility remains uncertain. Several *in vitro* exposures may not reflect achievable unbound concentrations in human plasma or tumour tissue. Their interpretation should therefore consider bioavailability, metabolism, formulation, tissue distribution and the distinction among dietary exposure, supplement dosing and purified-compound pharmacology.

### 7.5. Implications for Cancer Stem Cell Regulation, Recurrence and Resistance

Plant-derived bioactive compounds may modulate cellular senescence in a context-dependent manner. Some attenuate senescence-associated phenotypes or SASP signalling in non-malignant cells and tissues [123,124,126,127,128], whereas others induce senescence-associated growth arrest in cancer cells [129]. Modulation of inflammatory mediators such as IL-6 could potentially influence CSC-associated phenotypes, given the involvement of IL-6/STAT3 signalling in selected CSC models [65,94]. However, the compound studies reviewed in this section did not directly demonstrate that senescence modulation altered validated CSC functions, drug resistance or recurrence within the same experimental model [123,124,126,127,128,129]. These findings therefore provide mechanistic or preclinical plausibility rather than direct evidence of CSC regulation or recurrence prevention.

### 7.6. Risk Benefit Considerations

Modulation of cellular senescence requires consideration of both tumour control and normal tissue protection. Attenuating detrimental SASP signalling or eliminating persistent senescent cells may reduce treatment-associated damage and tumour-promoting effects in selected settings [6,54,97]. However, interventions that prevent or disrupt therapy-induced tumour-cell arrest could compromise its tumour-suppressive function [7,47]. Senescence escape may also expose enhanced stemness and tumour-propagating potential [91]. Importantly, senomorphic suppression of the SASP does not necessarily disrupt growth arrest [6,54]. Potential benefits and risks should therefore be assessed according to the mechanism of intervention, timing, target cell population, tumour genotype and treatment context [6,7].

### 7.7. Conclusion

**Figure 2:**
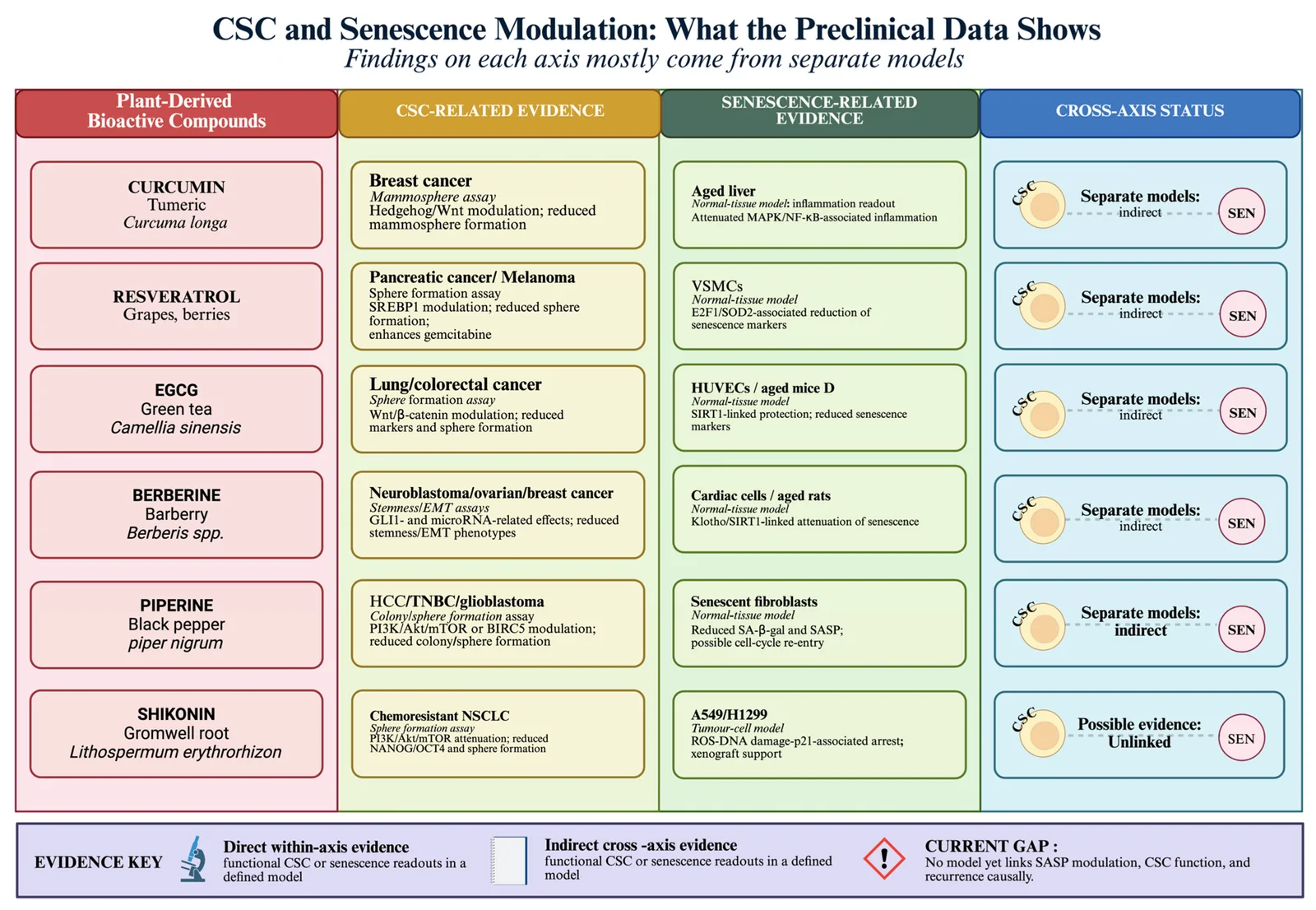
Preclinical evidence for CSC- and senescence-associated effects of selected plant-derived bioactive compounds. CSC-related and senescence-related findings are summarised by compound and experimental model. For most compounds, the two sets of findings come from separate studies; their juxtaposition does not demonstrate causal CSC–senescence crosstalk or prevention of recurrence. CSC, cancer stem cell; SASP, senescence-associated secretory phenotype; SEN, senescence. Created in BioRender. Naseer, S. (2026) https://app.biorender.com/illustrations/6aafacbc1406ada91d5fd514.

Collectively, plant-derived bioactive compounds can regulate senescence through redox control, MAPK/NF-κB signalling, E2F1/SOD2, and Klotho/SIRT1 pathways, but their biological value depends on cellular context. Curcumin, resveratrol, EGCG, berberine and piperine mainly provide preclinical evidence for attenuating pathological senescence or SASP-related stress in non-malignant cells, whereas shikonin illustrates the opposite strategy of inducing senescence in cancer cells through ROS-DNA damage-p21 signalling. For prevention of post-treatment recurrence, the most relevant future question is whether these interventions suppress protumorigenic SASP and the downstream IL-6/STAT3-CSC axis without weakening beneficial tumour-suppressive senescence. Studies combining cell-type-resolved models, durable growth-arrest assays, SASP profiling and recurrence or drug-resistance endpoints are therefore needed before clinical transition. The CSC-related and senescence-related findings for each compound are compared in Figure 2; their presentation side by side does not establish that a compound causally links the two processes in the same model.

## 8. Preclinical Therapeutic Implications and Translational Relevance

Cancer recurrence and drug resistance remain major obstacles to durable cancer control, but the mechanisms underlying these outcomes are heterogeneous and differ across tumour types, therapies, and clinical settings. Evidence reviewed in the preceding sections indicates that CSC-associated states may contribute to residual disease and therapeutic adaptation, while persistent therapy-induced senescence (TIS) and SASP-associated signalling may influence the tumour microenvironment in selected experimental models [130]. These processes should not be interpreted as universal or independently sufficient causes of recurrence; rather, they represent biologically plausible, potentially complementary mechanisms that may be relevant to treatment failure. This section considers the translational significance of targeting CSC-related phenotypes and harmful persistent TIS/SASP-associated processes, with particular emphasis on the distinction between preclinical evidence and clinically demonstrated benefit.

### 8.1. Why CSCs and Senescent Cells Matter Clinically

Although chemotherapy and radiotherapy eliminate many tumour cells, a residual population can remain viable after treatment. CSCs are clinically important within this population because they can self-renew, initiate tumour formation, and withstand therapeutic stress [130]. Their persistence may therefore contribute to tumour recurrence and treatment resistance.

Cancer therapy can also induce TIS. Stable cell cycle arrest may initially restrain tumour growth; however, persistent senescent tumour or stromal cells can release a SASP containing inflammatory cytokines and chemokines. In defined experimental contexts, persistent SASP may support CSC survival, EMT, and therapy resistance by remodelling the tumour microenvironment [93].

Accordingly, CSC-associated pathways and harmful persistent TIS/SASP signalling represent complementary targets for reducing treatment resistance and recurrence potential. Plant-derived bioactive compounds are being investigated as possible adjuvant strategies because they may influence multiple survival, stemness, and stress-response pathways. The following section summarises preclinical evidence; it does not establish clinical prevention of recurrence.

#### Other Mechanisms of Drug Resistance

CSCs represent only one component of cancer drug resistance. Other mechanisms include increased drug efflux, altered drug metabolism, enhanced DNA repair, avoidance of apoptosis, EMT, epigenetic adaptation, microenvironmental protection and immune evasion. ATP-binding cassette transporters, including P-glycoprotein/ABCB1, ABCC1 and ABCG2, can reduce intracellular drug concentrations, whereas changes in DNA-repair and apoptotic pathways may enable tumour cells to survive treatment-induced damage [131]. Hypoxia, stromal-cell interactions and survival signalling within the tumour microenvironment can further promote drug tolerance [132]. Immune-mediated mechanisms may also impair treatment responses by facilitating immune evasion and establishing an immunosuppressive microenvironment [133]. Although some of these mechanisms overlap with CSC-associated phenotypes, they are not specific to CSCs. CSC-associated drug tolerance should therefore be considered one component of the broader and heterogeneous process of cancer drug resistance.

The tumour microenvironment may further promote drug resistance through hypoxia, stromal-cell interactions and activation of survival signalling [132]. Immune-related mechanisms can also impair treatment responses by facilitating immune evasion and establishing an immunosuppressive tumour microenvironment [133]. Some of these mechanisms overlap with CSC-associated characteristics, including increased drug efflux, enhanced DNA repair, epithelial–mesenchymal transition and resistance to apoptosis. However, these mechanisms are not specific to CSCs. CSC-associated drug tolerance should therefore be regarded as one component of the broader and heterogeneous process of cancer drug resistance.

### 8.2. Preclinical Evidence for Chemosensitisation and Radiosensitisation

#### 8.2.1. Curcumin

Curcumin has shown preclinical chemosensitising potential in several cancer models. In doxorubicin-resistant breast cancer cells, curcumin inhibited NF-κB activation, reduced Bcl-2 expression, and helped restore doxorubicin sensitivity [134]. Curcumin also sensitised colorectal cancer cells to 5-fluorouracil and increased cisplatin-associated apoptosis in non-small-cell lung cancer models [135,136]. These studies support pathway-specific chemosensitisation *in vitro* and in preclinical models; clinical efficacy and recurrence reduction remain unconfirmed.

#### 8.2.2. Resveratrol

Resveratrol has shown preclinical potential to modulate drug-resistance pathways and recurrence-associated CSC properties. In glioblastoma cells, it increased oxidative stress and promoted apoptosis through TRPM2 activation, thereby enhancing the effect of paclitaxel [137]. In gastric cancer models, resveratrol reversed doxorubicin resistance in association with PTEN/Akt-mediated EMT suppression [138]. In lung cancer models, it reduced stemness-associated markers, including CD133, ALDH1A1, and Nanog, while suppressing Wnt/β-catenin signalling [139]. These molecular changes are associated with chemoresistance and recurrence-related CSC phenotypes, rather than direct evidence of recurrence prevention.

#### 8.2.3. EGCG

EGCG, a major green-tea polyphenol, has shown preclinical activity against multidrug resistance. In selected models, EGCG reduced MDR1/P-glycoprotein-associated drug efflux and enhanced intracellular exposure to chemotherapeutic agents [140]. It has also been associated with apoptosis and improved responses to doxorubicin, cisplatin, or tamoxifen in resistant cancer-cell models [126]. These data support a possible chemosensitising role but require validation in animal models and clinical studies.

#### 8.2.4. Berberine

Berberine has shown preclinical chemosensitising and radiosensitising activity in selected models. In cisplatin-resistant gastric cancer cells, berberine suppressed PI3K/Akt/mTOR signalling, reduced multidrug-resistance proteins, and restored cisplatin sensitivity [141]. In nasopharyngeal carcinoma cells, it enhanced radiation response in association with Sp1 and EMT inhibition [142]. These findings support treatment-sensitising potential in experimental models; they do not establish clinical benefit.

#### 8.2.5. Piperine

In triple-negative breast cancer models, piperine combined with doxorubicin produced greater tumour inhibition than doxorubicin alone. This effect was associated with PI3K/Akt/mTOR suppression, increased PTEN expression, and reduced ALDH1 expression [119]. In glioblastoma stem-cell models, piperine inhibited survivin and reduced SOX2, Nestin, and CD133 expression, findings consistent with reduced stemness-associated survival [120]. Together, these results support piperine as a potential chemosensitising compound in preclinical models.

Piperine requires particular caution because it can inhibit drug-metabolising enzymes and transporters, including CYP3A4 and P-glycoprotein [119]. Consequently, apparent chemosensitisation may partly result from increased intracellular or systemic exposure to the co-administered anticancer drug rather than selective modulation of CSC-associated pathways. Future combination studies should therefore incorporate pharmacokinetic analyses, transporter and enzyme assessments, and toxicity monitoring to distinguish pharmacological exposure effects from pathway-specific sensitisation.

#### 8.2.6. Shikonin

Shikonin has a distinct proposed role in overcoming drug resistance because it can induce necroptosis, a regulated cell-death pathway distinct from apoptosis. Shikonin analogues have shown activity against multidrug-resistant cancer cells expressing P-glycoprotein, MRP1, BCRP, and antiapoptotic proteins [143]. In addition, β, β-dimethylacrylshikonin enhanced the radiation response of colorectal cancer cells through ROS generation, DNA damage, and apoptosis after irradiation [144]. These studies suggest an alternative preclinical strategy for targeting therapy-resistant cells.

Improved treatment response should not automatically be interpreted as selective targeting of CSCs or drug-resistant populations. Reduced cell viability or tumour growth may reflect general cytotoxicity unless resistant and treatment-sensitive populations are compared under equivalent exposure conditions and the findings are supported by functional CSC assays, normal-cell controls or causal pathway interventions.

### 8.3. Phytochemicals, Senescence, CSCs and Immune-Checkpoint Therapy

Treatment response may be influenced when cellular senescence, CSCs and antitumour immunity interact with each other. During the early stage of therapy-induced senescence (TIS), senescent tumour cells can release SASP factors to recruit NK cells and CD4+ and CD8 T+ cells [52]. NK cells can recognise senescent cells with NKG2D receptors and contribute to immune-mediated clearance [62,73]. However, persistent senescent cells may evade immune clearance and contribute to an immunosuppressive tumour microenvironment [52]. Senescent cells may also show increased PD-L1 expression, which can contribute to immune evasion [86,87].

Next, persistent senescence may also interact with CSC-associated phenotypes. Senescence-associated signals can support stemness-related changes in selected experimental models [65]. These findings support that immune clearance of persistent senescent cells may be relevant to treatment response and resistance.

Plant-derived bioactive compounds may link these processes by affecting pathways involved in CSCs, inflammation, oxidative stress and also cell survival [9]. However, these effects do not directly show increased NK-cell or CD8+ T-cell activity, immune clearance or improved response to immune-checkpoint inhibitors. Thus, the interaction between phytochemicals, CSCs, senescence, and immune-checkpoint therapy remains a mechanistic and preclinical hypothesis. Future studies should examine these effects together with PD-L1/PD-L2 expression and immune-cell activity.

### 8.4. Translational Implications and Current Limitations

Current evidence for phytochemicals is derived predominantly from cell-culture and animal studies; therefore, evidence supporting their clinical use remains limited. Poor bioavailability, limited absorption, rapid metabolism, and variable pharmacokinetic properties may restrict the concentration of active compounds reaching tumour tissue [145]. Optimal dose, formulation, treatment schedule, drug–phytochemical interactions, and long-term safety also remain insufficiently defined. Well-designed clinical studies are required before these agents can be considered for routine use as adjuvant cancer therapies.

### 8.5. Take Home Message

Plant-derived bioactive compounds may complement conventional cancer therapy by targeting CSC-associated survival pathways and harmful persistent TIS/SASP signalling. Preclinical findings suggest that selected compounds can enhance responses to chemotherapy or radiotherapy and may modulate mechanisms associated with treatment resistance and recurrence. However, their effects are context-dependent, and most evidence is derived from cell-culture and animal studies [146,147]. Clinical applications will require evidence of safety, optimal dosing, bioavailability, drug-interaction profiles, and therapeutic benefit in well-designed clinical studies.

## 9. Translational Challenges and Research Priorities

**Figure 3:**
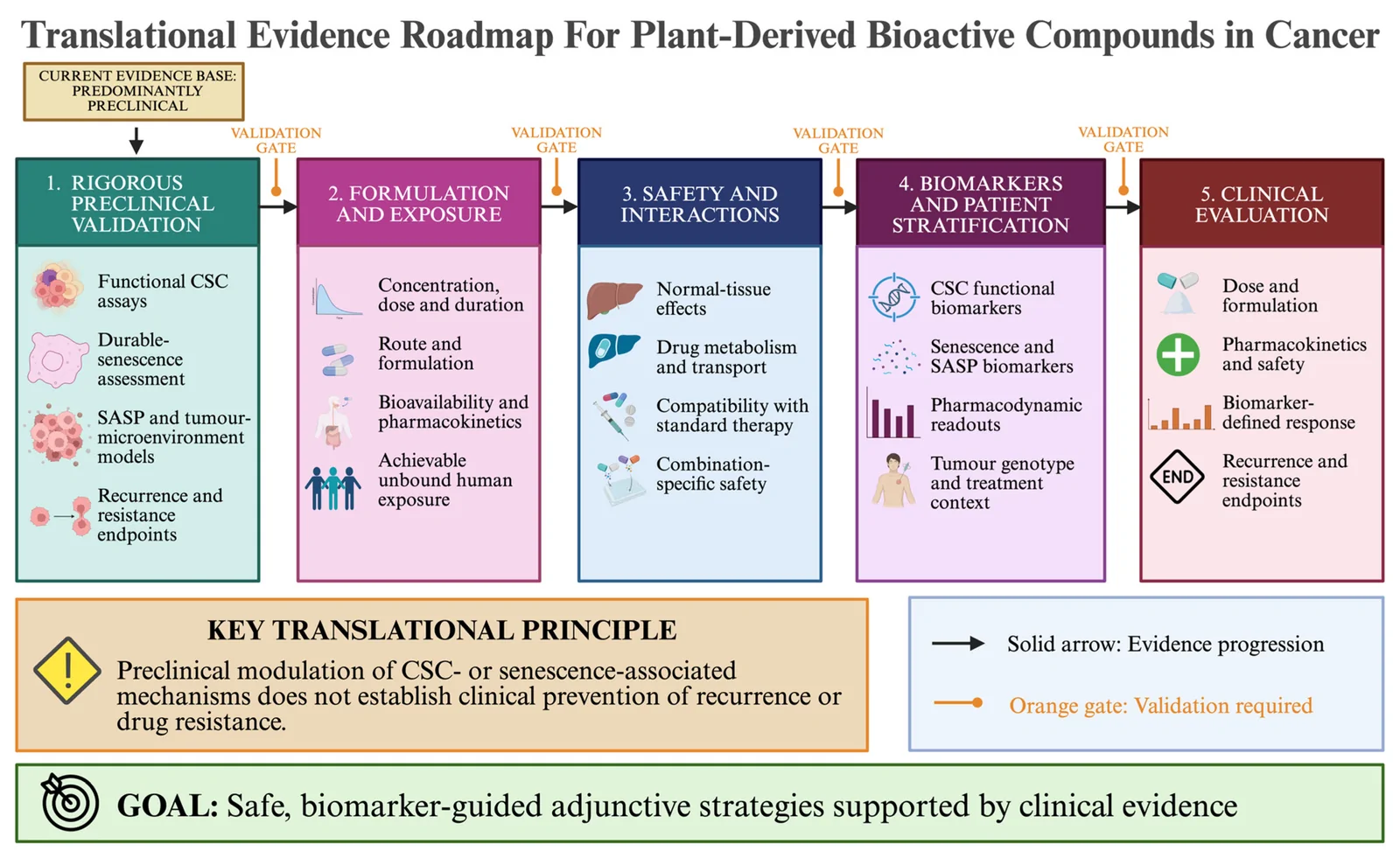
Translational evidence roadmap for plant-derived bioactive compounds in cancer. Progression from preclinical findings to clinical evaluation requires validation of functional effects, achievable exposure, safety, and biomarkers. Modulation of cancer stem cell (CSC) or senescence-associated pathways alone does not establish clinical prevention of recurrence or drug resistance. Created in BioRender. Naseer, S., Tan, W.Y, Bay, P.E (2026) https://app.biorender.com/illustrations/6ab0d6e6f86166a38934ba5a.

The preclinical evidence reviewed in the preceding sections suggests that plant-derived bioactive compounds may modulate CSC-related phenotypes, TIS, and persistent SASP-associated signalling. However, most studies examine these processes separately, and the available evidence does not establish that these compounds prevent recurrence or overcome drug resistance in patients. Selected clinical studies are summarised in Table 5. Their endpoints include tissue biomarkers, precancerous lesion recurrence and cancer diagnosis, but they do not directly establish CSC or senescence targeting in patients. Translation therefore requires a shift from compound-centred claims towards an evidence-based framework that integrates pharmacologically achievable exposure, target engagement, model relevance, safety, drug interactions, patient selection and clinically meaningful endpoints [148,149,150]. These sequential validation requirements, from functional preclinical evidence through exposure, safety, biomarker selection and clinical evaluation, are summarised in Figure 3.

### 9.1. Bioavailability, Exposure, and Formulation Are Central Challenges

Many phytochemicals display limited aqueous solubility, chemical instability, extensive first-pass metabolism, rapid elimination, and substantial interindividual variability in absorption. Consequently, an *in vitro* concentration that changes CSC-associated markers or senescence-related pathways may not be achieved safely in tumour tissue after oral administration. Plasma concentration alone is also insufficient: the relevant measure is the time-dependent, unbound exposure in the tumour and in normal tissues where a compound may have distinct effects on senescence and SASP [148,149,150].

Accordingly, nominal concentrations used in cell culture should be compared, where possible, with achievable unbound plasma and tumour concentrations in humans [149]. Studies should also distinguish exposure to the parent compound from exposure to active or inactive metabolites. Without such comparisons, biological activity observed at high *in vitro* concentrations cannot be assumed to have nutritional, pharmacological, or clinical relevance [149,150].

This issue is particularly apparent for curcumin. Early-phase studies of enhanced formulations suggest that systemic exposure can be increased without an obvious increase in adverse events, but clinical studies remain heterogeneous in formulation, dose, tumour type, co-treatment, and outcome measures. Co-administration with piperine has also increased curcumin exposure in healthy volunteers, illustrating the potential of a bioavailability enhancer. Nevertheless, a recent assessment of curcumin trials did not find convincing evidence of a significant therapeutic benefit in malignant disease. Increased exposure is therefore not, by itself, evidence of anticancer efficacy [149,151].

Piperine also warrants caution in combination strategies. Experimental studies show that it can inhibit CYP3A4 and P-glycoprotein, potentially altering exposure to co-administered anticancer drugs. Apparent chemosensitisation could therefore partly reflect a pharmacokinetic interaction rather than selective modulation of CSC-associated or senescence-related mechanisms. Future studies should report preparation composition and batch quality, dose rationale, pharmacokinetics, circulating metabolites, tissue distribution, drug interactions, and toxicity [152].

### 9.2. Nanoparticle-Enabled Delivery: Opportunity, Not a Substitute for Evidence

Lipid nanoparticles, polymeric nanoparticles, micelles, nanoemulsions, liposomes, phytosomes, and stimulus-responsive systems may improve the solubility, stability, circulation time, and cellular uptake of phytochemicals. Surface ligands or microenvironment-responsive release strategies may also help enrich delivery to selected tumour sites or CSC-associated populations. These approaches are attractive because they may simultaneously deliver a phytochemical and a conventional anticancer drug at a controlled ratio [153,154].

Nevertheless, nanoparticle delivery should not be assumed to produce tumour-specific exposure or clinical benefit. Biological barriers, heterogeneous vascular permeability, uptake by the mononuclear phagocyte system, manufacturing reproducibility, scale-up, long-term safety, and regulatory characterisation can all limit translation. The performance of a nanoformulation should therefore be demonstrated through biodistribution, pharmacokinetics, target engagement, and efficacy in clinically relevant models, rather than inferred from particle size or *ins vitro* cytotoxicity alone [153,154,155].

### 9.3. More Predictive Preclinical Models and Pharmacodynamic Biomarkers Are Needed

Conventional monolayer cell lines are useful for screening but cannot fully represent tumour heterogeneity, stromal interactions, immune surveillance, treatment-induced senescence, or the evolution of residual disease. Future studies should combine complementary models, including patient-derived organoids, co-cultures with fibroblasts or immune cells, patient-derived xenografts, and immunocompetent models when immune or SASP-mediated mechanisms are proposed. CSC-associated marker changes should be supported by functional endpoints, such as serial sphere formation, clonogenicity or limiting-dilution tumour initiation [156,157].

For studies proposing CSC–senescence crosstalk, both axes should be evaluated within the same experimental model, as illustrated by studies linking senescence or SASP-associated signals to functional stemness outcomes [65,91,93]. Such studies should combine validated CSC functional assays [17,20] with multiple complementary senescence markers, assessment of growth-arrest persistence after treatment withdrawal, proliferative-capacity measurements and SASP profiling [6,7,47,60,121]. Apoptosis and transient cell-cycle arrest should also be assessed as alternative explanations for reduced proliferation [6,121]. A causal intervention should then determine whether modifying senescence or the SASP alters CSC function, treatment response or residual disease [65,91,93,97].

Biomarker strategies should link the proposed mechanism to patient selection and early pharmacodynamic response. Depending on the hypothesis, useful biomarker panels may include cancer-type-appropriate CSC marker combinations together with sphere-forming or tumour-initiating capacity, TIS/SASP profiles, pathway activity, circulating tumour DNA, minimal residual disease, and immune-state measurements. The aim is not to identify one universal CSC or senescence marker but to define a reproducible, context-specific signature that can guide enrolment and interpret treatment effects [156,157].

### 9.4. Clinical Trial Design Should Test a Defined Adjunctive Hypothesis

Clinical development should prioritise well characterised formulations and a prespecified biological rationale rather than empiric supplementation [149,150,154,155]. Early studies should establish safety, pharmacokinetics, pharmacodynamic target engagement, and interaction with the standard regimen. Subsequent trials should evaluate a phytochemical as an adjunct to a defined chemotherapy, targeted therapy, radiotherapy, or immunotherapy backbone in a biologically selected population [149,150]. Randomisation, appropriate controls, adherence assessment, and predefined exposure-response analyses should be incorporated to strengthen interpretation.

Immune-based combination strategies require particular consideration. Senescent cells and the SASP can influence immune cell recruitment, immune clearance, immunosuppression and immune checkpoint signalling in a context-dependent manner [52,53,62,85,86,87,88,89]. These findings provide a rationale for investigating immune combinations but do not establish the compatibility of plant-derived bioactive compounds with immune-checkpoint inhibitors. Preclinical combination studies should therefore assess tumour cell and stromal senescence, immune cell composition, checkpoint expression, treatment sequence and toxicity before clinical benefit is inferred.

Endpoints should progress from mechanism-proximal measures to patient-relevant outcomes. Suitable early endpoints include tumour or blood biomarkers of pathway modulation, CSC-related functional changes, TIS/SASP signatures, and treatment tolerability [7,60,151,156]. Later studies should evaluate minimal residual disease, time to progression, recurrence-free survival, and overall survival when justified. Combination studies must also assess whether the intervention protects normal tissues while inadvertently preserving senescent tumour cells or whether it promotes tumour-cell senescence without adequate immune clearance [91,97,157]. Selected clinical studies of plant-derived bioactive compounds are summarised in Table 6, highlighting their endpoints, reported findings and limitations in establishing clinical CSC targeting or senescence modulation.

### 9.5. Translational Perspective

The most credible future role for plant-derived bioactive compounds is not as single-agent substitutes for established anticancer therapies but as investigated adjuncts directed towards a defined and pharmacologically tractable vulnerability. Potential applications include modulation of a CSC-associated survival programme, an adverse persistent-SASP microenvironment, a treatment-induced stress response, or another validated resistance mechanism. However, these applications should not be combined into a single therapeutic claim unless they have been demonstrated within the same model.

Translation requires continuity across experimental stages: the same compound, formulation and treatment schedule should be linked to pharmacologically achievable exposure, target engagement, functional biological effects and clinically relevant outcomes. Studies should also distinguish direct cross-axis evidence from intermediate mechanistic findings and marker- or pathway-level associations. This approach will help identify which compounds merit clinical evaluation and which laboratory observations are unlikely to translate [148,156,157].

## 10. Conclusions

CSCs and therapy-associated cellular senescence are biologically distinct but potentially interacting contributors to residual disease, treatment adaptation and recurrence-associated mechanisms. CSCs should be established using cancer-type-appropriate marker combinations supported by validated functional assays. Similarly, cellular senescence should be distinguished from transient arrest, quiescence, differentiation and cytotoxicity through multiple markers, persistence assessment, proliferative-capacity testing and SASP characterisation.

The central finding of this review is that evidence concerning plant-derived bioactive compounds and CSCs and evidence concerning these compounds and cellular senescence remain largely parallel. Few, if any, studies have demonstrated that the same compound, within the same cancer or tumour-microenvironment model, modifies both a validated CSC function and a defined senescence or SASP and subsequently reduces residual disease, treatment resistance or recurrence. Modulation of shared pathways or markers therefore represents indirect mechanistic support rather than proof of a functional CSC–senescence relationship.

Curcumin, resveratrol, EGCG, berberine, piperine and shikonin have demonstrated potentially relevant biological activities in preclinical models. However, the strength and meaning of these findings vary according to the compound, model, formulation, exposure and endpoint. Moreover, senescence induction, senomorphic activity and senolysis are mechanistically distinct strategies and may produce opposing effects depending on whether tumour cells, stromal cells or normal tissues are affected.

These compounds should therefore be regarded as investigational adjunctive candidates rather than established treatments for preventing recurrence or overcoming drug resistance. Future studies should use pharmacologically achievable exposures, standardised formulations, appropriate tumour–stroma–immune models, causal experimental designs and longitudinal outcomes. Clinical development should proceed only when target engagement, safety, drug interactions and a clearly defined adjunctive mechanism have been established.

## Figures and Tables

**Table 2:** CSC–senescence evidence and treatment outcomes. (**A**) Studies assessing a CSC–senescence relationship or a related cytokine–stemness mechanism. (**B**) Senescence-persistence and treatment outcomes without CSC assessment.

**Panel (A)**										
**Study**	**Model and Cell Source**	**Senescence Inducer or Assessed**	**Treatment Exposure: Concentration/Dose, Duration and Route**	**Senescence Verification and SASP Readouts**	**CSC-Associated Markers and Functional Endpoints**	**Causal Intervention**	**Source of Mediator or Mechanism**	***In Vivo*** **Validation**	**Evidence Hierarchy**	**Type of Evidence**
Ortiz-Montero et al. [65]	MCF-7 breast carcinoma	Yes	Senescence-conditioned medium for 5 days, refed every 2 days; recombinant IL-6/IL-8 at 50 ng/mL for 5 days, or 0.5 ng/mL for 10 days for autocrine senescence induction	Senescence-associated-β-galactosidase staining, p16/p21/p53 qRT-PCR; SCM confirmed enriched in IL-6/IL-8 by cytometric bead array	Functional—serial mammosphere self-renewal, multilineage differentiation	IL-6/IL-8 neutralising antibodies (loss): recombinant IL-6/IL-8 (gain)	Senescent-cell conditioned medium	None, all are *in vitro*	Direct cross-axis evidence	*In vitro* functional validation
Milanovic et al. [91]	Eμ-Myc B-cell lymphoma; T-ALL; AML; human lines and primary samples	Yes	Adriamycin (ADR), 5-day exposure; switchable genetic senescence models (Suv39h1-ERT2, p53-ERTam); γ-irradiation as alternative trigger	SA-β-gal, H3K9me3; adult tissue stem-cell (ATSC) transcript signature by GSEA	Functional—serial replating to p14; limiting-dilution tumour initiation *in vivo*	Suv39h1-deficient senescence-incapable control; Wnt inhibition (ICG-001, salinomycin); shCtnnb1	Senescent-cell intrinsic (SASP-independent; persists under NF-κB blockade)	*In vivo* tumour-initiation assays; nuclear β-catenin enrichment observed in relapsed versus primary murine lymphomas; correlative human biopsy findings	Intermediate mechanistic evidence	*In vitro* and *in vivo* functional validation, correlative human data
Karabicici et al. [92]	HuH7 HCC; sorted EpCAM^+^/CD133^+^ and EpCAM^−^/CD133^−^ cells	Yes	Doxorubicin 100 nM, 48 h, followed to day 15	SA-β-gal; p16/p21/p53; IL-6/TGF-β1 qPCR; IL-8 and IP-10 by ELISA	Stemness genes; EpCAM^+^ fraction; NSG tumour incidence	IWR-1 (chemical Wnt inhibition)	Cell-intrinsic	Yes, NSG xenografts of bulk Dox-treated cells; no recurrence endpoint	Direct cross-axis evidence	*In vitro* and *in vivo* functional validation
Cahu et al. [93]	Multiple myeloma	Yes	X-irradiation (6 Gy), assessed 7 days later; doxorubicin (50 nM), assessed 2 days later	β-galactosidase/C12FDG staining, γH2AX, sustained CHK2 phosphorylation; cytokine array and qRT-PCR identifying IP-10 and RANTES	Functional—emergence of stem-like cells; CD138low population	Conditioned-medium transfer; CHK2 knockdown	Senescent-cell conditioned medium	Yes—xenograft tumour formation assessed in nude mice; the causal SASP/CHK2 intervention was not validated against an *in vivo* recurrence endpoint	Direct cross-axis evidence	*In vitro* and *in vivo* functional validation
TAM-derived IL-6 [94]	Breast carcinoma	No	Conditioned medium (CM) from 4T1-activated RAW264.7 macrophages, 24 h *in vitro*; intratumoral injection of CM every other day for 2 weeks *in vivo*	None—no senescence measured anywhere in this study	Functional—ALDH1, Sca-1, mammosphere formation	IL-6 neutralisation; STAT3 inhibition	Non-senescent cell-derived cytokine (macrophage)	Yes—orthotopic 4T1 tumours in BALB/c mice; intratumoral CM injection increased tumour volume/weight and CSC population (Sca-1+ cells sorted from tumours, 8.5% vs 3.1% control), reversed by IL-6 neutralisation	Intermediate mechanistic evidence	*In vitro* and *in vivo* functional validation
Recombinant IL-8 [95]	H446 small-cell lung carcinoma	No	IL-8 (10 ng/mL for sphere assays); siRNA knockdown (siIL-8, 30 nM)	None (senescence not induced or measured)	Functional—tumoursphere formation	IL-8 silencing	Recombinant cytokine	Yes, but for the IL-8 to CSC axis only, not senescence—intratumoral siIL-8 injection reduced xenograft tumour growth	Intermediate mechanistic evidence	*In vitro* and *in vivo* functional validation
Continuous TGF- β [96]	Breast and skin carcinoma	No	Continuous TGF-β1 (5 ng/mL), 7 days in 2D followed by 5 days in 3D culture	None (senescence not induced or measured)	Functional—intermediate EMT state, stemness phenotypes	Continuous ligand exposure	Recombinant exposure	Yes—orthotopic transplantation, tumour initiation and metastasis assessed	Intermediate mechanistic evidence	*In vitro* and *in vivo* functional validation
**Panel (B)**										
**Study**		**Model**		**Senescence assessed**	**Outcome measured**		**Causal intervention**		**Evidence and limitation**	
PD-L2 [89]		Pancreatic, mammary tumours		Yes	Chemotherapy efficacy		PD-L2 deficiency		Animal treatment response; no CSC readout	
Senescent-cell clearance [97,98]		Murine breast cancer		Yes	Metastatic burden; regrowth delay		Elimination of host senescent cells		Recurrence-related animal outcome; no CSC readout	

**Table 4:** Summary of the effects of selected bioactive compounds on cellular senescence and their potential relevance to cancer.

Plant-Derived Bioactive Compounds	Model	Experimental Exposure (Concentration or Dose and Treatment Duration)	Administration and Formulation (Administration on Route, Delivery Method and Formulation/Vehicle)	Direction	Key Pathway	Senescence Evidence Category and Supporting Evidence	Functional Validation	*In Vivo* Evidence	Cancer Relevance	Main Limitation
**Curcumin** [123]	Aged mouse liver; primary hepatocytes	*In vitro*: 10 µM for 24 h; *in vivo*: 0.4% (*w*/*w*) curcumin for 6 and 15 weeks	Oral administration; incorporated into diet	Attenuates hepatic senescence-associated inflammation	MAPK/NF-κB	Senescence-associated inflammatory modulation; senomorphic classification not conclusively established. Curcumin reduced the expression of inflammatory genes and the p-p65/p65 ratio, but direct modification of an established senescent phenotype was not demonstrated	No direct senescence-specific functional validation	Non-malignant aged hepatic tissue; no tumour model	Possible SASP/microenvironment relevance	No direct tumour cell, CSC, recurrence, or resistance endpoint
**Resveratrol** [126]	Ang II VSMCs	*In vitro:* VSMCs were exposed to 100 nM Ang II for 3 days, followed by resveratrol (0.5, 5, 15, 25, 35 or 50 µM) for 24 h; 25 µM was selected for subsequent experiments	*In vitro* addition to culture medium; formulation or vehicle not reported	Attenuates senescence	E2F1/SOD2	Senescence-associated phenotype + mechanistic validation: SA-β-gal; p21/p53/γ-H2AX; knockdown	E2F1 knockdown supports role of the E2F1/SOD2 pathway in regulating senescence	No *in vivo* validation	Possible microenvironment relevance	Non-cancer model; no recurrence endpoint
**EGCG** [127]	DOX HUVECs; aged mice	*In vitro*: doxorubicin-treated human umbilical vein endothelial cells (HUVECs); *in vivo*: naturally aged mice	*In vitro* cell exposure; vehicle not reported; *in vivo*: oral gavage	Protects non-malignant endothelial cells against doxorubicin-induced senescence	SIRT1–autophagy/ferroptosis	Protection against senescence induction in non-malignant endothelial cells, supported by reduced SA-β-gal activity and lower expression of p53, p21 and p16. SIRT1 inhibition and knockdown attenuated these protective effects, supporting mechanistic involvement of SIRT1	SIRT1 inhibition attenuated the protective effect of EGCG	Naturally aged mice; vascular ageing was assessed without doxorubicin treatment	Normal-tissue protection only	No direct cancer/CSC evidence
**Berberine** [128]	DOX H9c2 cells; aged rats	*In vitro*: doxorubicin-treated H9c2 cardiac cells; *in vivo*: naturally aged rats	Oral administration via drug-containing diet	Attenuates doxorubicin-associated senescence in H9c2 cells and age-associated cardiac senescence in rats	Klotho/SIRT1	Protection against doxorubicin-associated senescence in non-malignant cardiac cells, supported by reduced SA-β-gal activity and lower p21 and p53 expression. Klotho knockdown attenuated these protective effects, supporting the involvement of the Klotho/SIRT1 pathway	Klotho knockdown attenuated protective effect of berberine	Naturally aged rat model; doxorubicin was not administered to the rats	Age-associated senescence relevance	No cancer-associated senescence/CSC endpoint
**Piperine** [124]	Premature S-HDFs	*In vitro*: 70 µM three times at 2-day intervals	*In vitro* cell exposure, added to complete culture medium	Modulates senescence-associated phenotypes; possible cell-cycle re-entry or escape	ERK/JNK-related	Modulation of senescence-associated phenotypes in non-malignant fibroblasts, supported by reduced SA-β-gal activity, SASP mediators and ROS, together with improved mitochondrial membrane potential. However, the increased division of previously senescent cells may indicate cell-cycle re-entry or senescence escape rather than beneficial reversal of senescence	Increased division of senescent S-HDFs; durability of growth arrest was not assessed	No *in vivo* validation of the senescence-modulating effects reported	Potential modulation of senescence-associated phenotypes and SASP relevance	Re-entry and long-term safety unclear
**Shikonin** [129]	Lung cancer cells; xenografts	*In vitro:* shikonin at 1 and 2 μM (A549) or 0.3 and 0.6 μM (H1299) for 72 h. *In vivo:* shikonin at 5 and 10 mg/kg (A549); 10 mg/kg (H1299)	*In vitro*: cell exposure; vehicle not reported. *In vivo*: oral gavage, sterilised corn oil used as the gavage control; SHK formulation/vehicle not explicitly stated	Induces senescence-associated growth arrest in tumour cells	ROS–DNA damage–p21	Senescence induction was supported by SA-β-gal, morphological changes, γ-H2AX, p21, reduced EdU incorporation and G0/G1 arrest in both cell lines, with corroborating xenograft findings. p53 contributed in A549 cells, whereas p21 induction was p53-independent in H1299 cells. Durability and the contribution of apoptosis were not established	↓ EdU; G0/G1 arrest; ↓ cyclin D1 (cell models)	↓ tumour weight	Potential anticancer relevance	Durability, escape, CSC persistence, recurrence and resistance not tested

**Note:** ↓ indicates a decrease in the measured parameter (EdU fluorescence, cyclin D1 expression, tumour weight) following shikonin treatment compared with the control.

**Table 5:** Preclinical evidence for treatment sensitisation and CSC-associated effects of selected plant-derived bioactive compounds.

Compound	Cancer Model/Combination Treatment	Experimental Concentration or Dose	Treatment Duration	Administration Route	Formulation or Vehicle	Treatment-Response Evidence	Drug-Resistant/CSC-Related Evidence	Functional Endpoint Reported	*In Vivo* Validation	Resistance or Recurrence Endpoint	Evidence Stage
Curcumin [134,135,136]	-Breast cancer + doxorubicin -5-FU-resistant colorectal cancer -Cisplatin-resistant NSCLC	5-FU-resistant CRC: curcumin 5–20 μM 5-FU 0.1–5 μM	CRC: 1–10 days for colonosphere experiments 24 h for some sensitivity assays	*In vitro*	CRC: culture-based treatment	Restored doxorubicin and 5-FU sensitivity Increased cisplatin sensitivity	Doxorubicin-, 5-FU- and cisplatin-resistant models CSC-specific selectivity not established	Cell viability, apoptosis, colonosphere formation in the 5-FU-resistant model	Doxorubicin-resistant Ehrlich ascites carcinoma–bearing mice: curcumin plus doxorubicin reduced viable tumour cells and improved survival compared with doxorubicin treatment alone. No recurrence endpoint was assessed	Drug-resistance reversal was assessed Recurrence was not directly measured	Preclinical chemosensitisation
Resveratrol [137,138,139]	-Glioblastoma + paclitaxel -Doxorubicin-resistant gastric cancer -Lung cancer stem-like cells	Glioblastoma: resveratrol 50 μM + paclitaxel 50 μM. Gastric cancer: 20–200 mg/L resveratrol; combination experiments included 50 mg/L. Lung CSC model: study-specific	Glioblastoma: 24 h. Gastric cancer: treatment periods varied; 48 h for some molecular analyses	*In vitro*; animal administration for xenograft study	Study-specific	Enhanced paclitaxel response Restored doxorubicin sensitivity Suppressed lung cancer stem-like properties	Doxorubicin-resistant cells CSC-associated markers and stem-like model	Cell viability, apoptosis, colony formation, tumour growth Tumoursphere formation in the lung CSC model	Tumour growth inhibition reported in animal models	Doxorubicin resistance reversal was assessed; recurrence was not directly measured	Preclinical chemosensitisation and CSC-associated functional effects
EGCG [126,140]	-5-FU-resistant gastric cancer -multidrug-resistant esophageal cancer	Gastric cancer: 20 μM EGCG for MDR1/P-gp experiments	Gastric cancer: 48 h for MDR1/P-gp analysis; 30 days for xenograft treatment	*In vitro:* added to culture medium. *In vivo:* the gastric xenograft study reports intratumoral injection of the PBS control but does not clearly specify the administration route for EGCG and 5-FU; do not label their route as oral	Study-specific	In 5-FU-resistant gastric cancer cells, EGCG increased sensitivity to 5-FU; EGCG plus 5-FU also reduced growth of resistant gastric cancer xenografts. Separately, EGCG reduced multidrug resistance and promoted apoptosis in oesophageal cancer cells	5-FU-resistant cells ABCG2-mediated multidrug-resistant cells Reduced MDR1/P-gp or ABCG2	Cell proliferation, viability, apoptosis, intracellular drug accumulation Xenograft tumour growth	Xenograft tumour growth inhibition with EGCG + 5-FU	Drug resistance reversal was assessed; recurrence was not directly measured	Preclinical chemosensitisation/resistance reversal
Berberine [141,142]	-Cisplatin-resistant gastric cancer -Nasopharyngeal carcinoma + radiation	Gastric cancer: berberine 10 or 30 µM enhanced cisplatin sensitivity in resistant cells; mechanistic co-treatment used 30 µM berberine and 30 µM cisplatin. Xenografts received berberine 10 mg/kg/day with cisplatin 3 mg/kg/day. Nasopharyngeal carcinoma: berberine 25–100 µM combined with 4–8 Gy irradiation	Gastric cancer: 24 h for cell co-treatment; 30 days for xenograft treatment. Nasopharyngeal carcinoma: the radiosensitivity results describe 3 h of berberine pretreatment, followed by viability assessment 72 h after irradiation	*In vitro*; animal administration in gastric cancer model	Study-specific	Increased cisplatin sensitivity Enhanced radiosensitivity	Cisplatin-resistant cells Multidrug-resistance proteins Radioresistance model	Cell viability, apoptosis, caspase activity, cell-cycle arrest, radiosensitivity In vivo cisplatin response	*In vivo* cisplatin response reported	Cisplatin resistance/radiosensitivity was assessed; recurrence was not directly measured	Preclinical chemo-/radiosensitisation in resistant models
Piperine [119,120]	TNBC + doxorubicin; glioblastoma cancer stem cells + temozolomide	TNBC: piperine 100 or 200 μM with doxorubicin. GSC: 25 μM piperine for survivin analysis; concentrations below IC50 tested with TMZ	GSC: 48 h for survivin analysis; combination effects assessed at 24, 48 and 72 h. TNBC: study-specific	*In vitro*; animal model for TNBC	GSC: ethanol/DMSO vehicle controls reported; TNBC: study-specific	Enhanced doxorubicin and temozolomide responses	CSC-associated ALDH-1 in TNBC; direct GSC model in glioblastoma	Cell viability, tumour volume/survival, self-renewal, proliferation, apoptosis and drug response	Tumour response in TNBC animal model	Treatment sensitisation was assessed; recurrence was not directly measured	Preclinical treatment sensitisation with CSC-associated effects; stronger CSC-focused evidence, but selective CSC targeting should not be assumed
Shikonin [143,144]	Drug-resistant cancer cells; colorectal cancer + ionising radiation	*In vitro:* shikonin or its analogue β, β-dimethylacrylshikonin, 0.2–1 µM in the HCT-116 radiosensitisation experiments. *In vivo:* 2.0 mg/kg shikonin or analogue in HCT-116 xenograft-bearing mice	*In vitro:* added to cell culture before irradiation. *In vivo:* administered by injection; the paper does not clearly specify the injection route	Drug-resistant cancer cells; colorectal cancer + ionising radiation	*In vivo* control vehicle: 10% DMSO in PBS	β, β-Dimethylacrylshikonin enhanced the response of HCT-116 cells to ionising radiation and, when combined with irradiation, suppressed HCT-116 xenograft growth	Drug-resistant cancer cells; colorectal cancer + ionising radiation	Necroptosis/cytotoxicity, apoptosis, ROS, DNA damage, cell viability, xenograft tumour growth	Xenograft tumour growth suppression with β,β-dimethylacrylshikonin + radiation	Drug resistance and radiosensitivity were assessed; recurrence was not directly measured	Preclinical resistance-associated cytotoxicity and radiosensitisation

**Table 6:** Selected clinical studies of plant-derived bioactive compounds in oncology.

Compound and Study	Cancer Type or Study Population	Phase and Status	Primary Endpoint or Main Assessment	Reported Results and Relevance to This Review	Reference
Curcumin + FOLFOX; NCT01490996	Metastatic colorectal cancer	Phase IIa; completed	Safety and tolerability of adding oral curcumin to FOLFOX	The combination was reported as safe and tolerable. The study does not establish that curcumin prevents recurrence or targets CSCs in patients.	[158]
Resveratrol or resveratrol-containing grape powder; NCT00256334	Patients with colon cancer	Phase I pilot; completed	Wnt-pathway target-gene expression in colonic tissue	Wnt target-gene expression decreased in normal colonic mucosa, but the study did not find Wnt-pathway inhibition in colon cancer tissue. This is tissue biomarker evidence, not evidence of improved cancer outcomes.	[159]
Green-tea catechins containing EGCG (Polyphenon E)	Men with high-grade prostatic intraepithelial neoplasia and/or atypical small acinar proliferation	Phase II; completed	Prostate cancer diagnosis over one year	The randomised trial found no statistically significant difference in prostate cancer rates between groups. This was a prevention study in a high-risk population, not a test of CSC targeting or cancer recurrence.	[160]
Berberine versus placebo; NCT02226185	Patients after removal of colorectal adenomas	Randomised controlled chemoprevention trial; completed	Colorectal adenoma recurrence at follow-up colonoscopy	Recurrent adenomas were reported in 36% of the berberine group and 47% of the placebo group. Adenoma recurrence is not recurrence of established colorectal cancer; the trial did not test CSC or senescence endpoints.	[161]
Curcumin + piperine; NCT02598726	Patients with cancer experiencing ureteral stent symptoms	Phase I pilot; completed	Safety, dose, and stent-related symptoms	No study results were posted in the registry record checked. This supportive-care study did not test anticancer efficacy.	[162]

## Data Availability

No new data were created or analyzed in this study. Data sharing is not applicable to this article.

## Abbreviations

The following abbreviations are used in this manuscript:

Abbreviation	Full meaning
CSC/CSCs	Cancer stem cell(s)
EMT	Epithelial–mesenchymal transition
EpCAM	Epithelial cell adhesion molecule
EpICD	EpCAM intracellular domain
LGR5	Leucine-rich repeat-containing G-protein-coupled receptor 5
PROM1	Prominin-1 (CD133 gene)
ALDH	Aldehyde dehydrogenase
ALDH1A1	Aldehyde dehydrogenase 1 family member A1
ALDH1A3	Aldehyde dehydrogenase 1 family member A3
OCT4	Octamer-binding transcription factor 4
POU5F1	POU class 5 homeobox 1
SOX2	SRY-box transcription factor 2
NANOG	NANOG homeobox transcription factor
MDR1	Multidrug resistance protein 1 (ABCB1)
SASP	Senescence-associated secretory phenotype
TIS	Therapy-induced senescence
SA-β-gal	Senescence-associated β-galactosidase
SAHF	Senescence-associated heterochromatin foci
senCAF/senCAFs	Senescent cancer-associated fibroblast(s)
PI3K	Phosphoinositide 3-kinase
AKT	Protein kinase B
mTOR	Mechanistic target of rapamycin
MAPK	Mitogen-activated protein kinase
MAPKs	Mitogen-activated protein kinases
NF-κB	Nuclear factor kappa B
STAT3	Signal transducer and activator of transcription 3
JAK	Janus kinase
JNK	c-Jun N-terminal kinase
ERK1/2	Extracellular signal-regulated kinase 1/2
SIRT1	Sirtuin 1
ROS	Reactive oxygen species
DDR	DNA-damage response
ATM	Ataxia-telangiectasia mutated
ATR	ATM and Rad3-related
MDM2	Mouse double minute 2 homolog
CDK4/6	Cyclin-dependent kinase 4 and 6
Rb/RB1	Retinoblastoma protein/Retinoblastoma 1
E2F	E2F transcription factor family
TCF	T-cell factor
LEF	Lymphoid enhancer-binding factor
CSL	CBF1/Suppressor of Hairless/LAG-1 transcription factor complex
RBPJ	Recombination signal binding protein for immunoglobulin kappa J region
NICD	Notch intracellular domain
HES	Hairy and enhancer of split family
HEY	HES-related family
Shh	Sonic Hedgehog
PTCH	Patched receptor
SMO	Smoothened receptor
GLI	GLI family zinc-finger transcription factors
APC	Adenomatous polyposis coli
CK1α	Casein kinase 1 alpha
SCF	SKP1–Cullin–F-box E3 ubiquitin ligase complex
RNF43	Ring finger protein 43
ZNRF3	Zinc and ring finger 3
IL-6	Interleukin-6
IL-8	Interleukin-8
TNF-α	Tumour necrosis factor alpha
TGF-β	Transforming growth factor beta
VEGF	Vascular endothelial growth factor
CXCL1	C-X-C motif chemokine ligand 1
CXCL2	C-X-C motif chemokine ligand 2
CXCL8	C-X-C motif chemokine ligand 8 (IL-8)
CXCL10	C-X-C motif chemokine ligand 10
CXCL12	C-X-C motif chemokine ligand 12
CXCR1/2	C-X-C chemokine receptors 1 and 2
DAMPs	Damage-associated molecular patterns
MMPs	Matrix metalloproteinases
EVs	Extracellular vesicles
TME	Tumour microenvironment
NK cells	Natural killer cells
NKG2D	Natural killer group 2 member D
PD-L1	Programmed death-ligand 1
PD-L2	Programmed death-ligand 2
MRP1	Multidrug resistance-associated protein 1
BCRP	Breast cancer resistance protein
PTEN	Phosphatase and tensin homolog
BIRC5	Baculoviral IAP repeat-containing protein 5 (survivin)
NSCLC	Non-small-cell lung cancer
HCC	Hepatocellular carcinoma
TNBC	Triple-negative breast cancer
HUVECs	Human umbilical vein endothelial cells
VSMCs	Vascular smooth muscle cells
S-HDFs	Senescent human diploid fibroblasts
DOX	Doxorubicin
5-FU	5-fluorouracil
ΔΨm	Mitochondrial membrane potential
γ-H2AX	Phosphorylated histone H2AX
p-p65/p65	Phosphorylated p65/total p65 NF-κB subunit ratio
EX527	SIRT1 inhibitor EX527
siRNA	Small interfering RNA
EGCG	Epigallocatechin gallate

## References

[1] Bray F., Laversanne M., Sung H., Ferlay J., Siegel R.L., Soerjomataram I., Jemal A. (2024). Global cancer statistics 2022: GLOBOCAN estimates of incidence and mortality worldwide for 36 cancers in 185 countries. CA Cancer J. Clin..

[2] Brown J.S., Amend S.R., Austin R.H., Gatenby R.A., Hammarlund E.U., Pienta K.J. (2023). Updating the Definition of Cancer. Mol. Cancer Res..

[3] Vasan N., Baselga J., Hyman D.M. (2019). A view on drug resistance in cancer. Nature.

[4] Li Y., Rogoff H.A., Keates S., Gao Y., Murikipudi S., Mikule K., Leggett D., Li W., Pardee A.B., Li C.J. (2015). Suppression of cancer relapse and metastasis by inhibiting cancer stemness. Proc. Natl. Acad. Sci. USA.

[5] Campisi J. (2013). Aging, cellular senescence, and cancer. Annu. Rev. Physiol..

[6] Di Micco R., Krizhanovsky V., Baker D., d’Adda di Fagagna F. (2021). Cellular senescence in ageing: From mechanisms to therapeutic opportunities. Nat. Rev. Mol. Cell Biol..

[7] Prasanna P.G., Citrin D.E., Hildesheim J., Ahmed M.M., Venkatachalam S., Riscuta G., Xi D., Zheng G., Deursen J.V., Goronzy J. (2021). Therapy-Induced Senescence: Opportunities to Improve Anticancer Therapy. J. Natl. Cancer Inst..

[8] Fong D., Chan M.M. (2013). Dietary Phytochemicals Target Cancer Stem Cells for Cancer Chemoprevention. Mitochondria as Targets for Phytochemicals in Cancer Prevention and Therapy.

[9] Chan M.M., Chen R., Fong D. (2018). Targeting cancer stem cells with dietary phytochemical—Repositioned drug combinations. Cancer Lett..

[10] Bao B., Ahmad A., Azmi A.S., Ali S., Sarkar F.H. (2013). Overview of cancer stem cells (CSCs) and mechanisms of their regulation: Implications for cancer therapy. Curr. Protoc. Pharmacol..

[11] Zhu Y., Chen S., Su H., Meng Y., Zang C., Ning P., Hu L., Shao H. (2025). CPT1A-mediated MFF succinylation promotes stemness maintenance in ovarian cancer stem cells. Commun. Biol..

[12] Speigl L., Janssen N., Weide B., Sinnberg T., Pawelec G., Shipp C. (2023). Putative Cancer Stem Cell Markers are Frequently Expressed by Melanoma Cells in Vitro and in Situ but are also Present in Benign Differentiated Cells. Front. Biosci..

[13] Zhang L., Chen W., Liu S., Chen C. (2023). Targeting Breast Cancer Stem Cells. Int. J. Biol. Sci..

[14] Zhang C., Du Z., Gao Y., Lim K.S., Zhou W., Huang H., He H., Xiao J., Xu D., Li Q. (2024). Methionine secreted by tumor-associated pericytes supports cancer stem cells in clear cell renal carcinoma. Cell Metab..

[15] Saha S., Parte S., Roy P., Kakar S.S. (2021). Ovarian Cancer Stem Cells: Characterization and Role in Tumorigenesis. Adv. Exp. Med. Biol..

[16] Fernando W., Cruickshank B.M., Arun R.P., MacLean M.R., Cahill H.F., Morales-Quintanilla F., Dean C.A., Wasson M.D., Dahn M.L., Coyle K.M. (2024). ALDH1A3 is the switch that determines the balance of ALDH^+^ and CD24^−^CD44^+^ cancer stem cells, EMT-MET, and glucose metabolism in breast cancer. Oncogene.

[17] Herreros-Pomares A., de-Maya-Girones J.D., Calabuig-Fariñas S., Lucas R., Martínez A., Pardo-Sánchez J.M., Alonso S., Blasco A., Guijarro R., Martorell M. (2019). Lung tumorspheres reveal cancer stem cell-like properties and a score with prognostic impact in resected non-small-cell lung cancer. Cell Death Dis..

[18] Kim S.J., Owen S.C. (2020). Hyaluronic acid binding to CD44S is indiscriminate of molecular weight. Biochim. Biophys. Acta Biomembr..

[19] Sihombing U.H.M., Andrijono A., Purwoto G., Gandamihardja S., Harahap A.R., Rustamadji P., Kekalih A., Widyawati R., Fuady D.R. (2022). CD44^+^/CD24^−^ Expression as predictors of ovarian cancer chemoresistance: Immunohistochemistry and flow cytometry study. J. Egypt. Natl. Cancer Inst..

[20] Curley M.D., Therrien V.A., Cummings C.L., Sergent P.A., Koulouris C.R., Friel A.M., Roberts D.J., Seiden M.V., Scadden D.T., Rueda B.R. (2009). *CD133* expression defines a tumor initiating cell population in primary human ovarian cancer. Stem Cells.

[21] Lien H.E., Hjelmeland M.E., Berg H.F., Gold R.M., Woie K., Akslen L.A., Haldorsen I.S., Krakstad C. (2025). Multiplex single-cell profiling of putative cancer stem cell markers ALDH1, SOX9, SOX2, CD44, CD133 and CD15 in endometrial cancer. Mol. Oncol..

[22] Aglan H.A., Abd-Rabou A.A., Ahmed H.H., Elsayed G.H., Kishta M.S., Elhinnawi M.A., Mahmoud N.S. (2025). Role of CD133 antibody-conjugated nanocarrier in enhancing the targetability of hepatocellular carcinoma stem cells. Sci. Rep..

[23] Li W., Ma H., Zhang J., Zhu L., Wang C., Yang Y. (2017). Unraveling the roles of CD44/CD24 and ALDH1 as cancer stem cell markers in tumorigenesis and metastasis. Sci. Rep..

[24] Chang P.M., Chen C.H., Yeh C.C., Lu H.J., Liu T.T., Chen M.H., Liu C.Y., Wu A.T.H., Yang M.H., Tai S.K. (2018). Transcriptome analysis and prognosis of ALDH isoforms in human cancer. Sci. Rep..

[25] Martinez-Cruzado L., Tornin J., Santos L., Rodriguez A., García-Castro J., Morís F., Rodriguez R. (2016). Aldh1 Expression and Activity Increase During Tumor Evolution in Sarcoma Cancer Stem Cell Populations. Sci. Rep..

[26] Zhang J., Qi Y.P., Ma N., Lu F., Gong W.F., Chen B., Ma L., Zhong J.H., Xiang B.D., Li L.Q. (2020). Overexpression of Epcam and CD133 Correlates with Poor Prognosis in Dual-phenotype Hepatocellular Carcinoma. J. Cancer.

[27] Thimsen V., Hölsken A., Buchfelder M., Flitsch J., Fahlbusch R., Stefanits H., Losa M., Jones D.T., Buslei R. (2016). EpCAM (CD326) is differentially expressed in craniopharyngioma subtypes and Rathke’s cleft cysts. Sci. Rep..

[28] Binnerts M.E., Kim K.A., Bright J.M., Patel S.M., Tran K., Zhou M., Leung J.M., Liu Y., Lomas WE3rd Dixon M., Hazell S.A. (2007). R-Spondin1 regulates Wnt signaling by inhibiting internalization of LRP6. Proc. Natl. Acad. Sci. USA.

[29] Dame M.K., Attili D., McClintock S.D., Dedhia P.H., Ouillette P., Hardt O., Chin A.M., Xue X., Laliberte J., Katz E.L. (2018). Identification, isolation and characterization of human LGR5-positive colon adenoma cells. Development.

[30] Shimokawa M., Ohta Y., Nishikori S., Matano M., Takano A., Fujii M., Date S., Sugimoto S., Kanai T., Sato T. (2017). Visualization and targeting of LGR5^+^ human colon cancer stem cells. Nature.

[31] Ding Y., Yuan X., Zou Y., Gao J., Xu X., Sun H., Zuo Q., Zhang Y., Li B. (2022). OCT4, SOX2 and NANOG co-regulate glycolysis and participate in somatic induced reprogramming. Cytotechnology.

[32] Gruszka O., Żelaszczyk D., Marona H., Bednarek I.A. (2025). Sensitisation of HeLa Cell Cultures to Xanthone Treatment by RNAi-Mediated Silencing of NANOG and STAT3. Curr. Issues Mol. Biol..

[33] Cho Y.H., Ro E.J., Yoon J.S., Mizutani T., Kang D.W., Park J.C., Il Kim T., Clevers H., Choi K.Y. (2020). 5-FU promotes stemness of colorectal cancer via p53-mediated WNT/β-catenin pathway activation. Nat. Commun..

[34] Raghavan S., Mehta P., Xie Y., Lei Y.L., Mehta G. (2019). Ovarian cancer stem cells and macrophages reciprocally interact through the WNT pathway to promote pro-tumoral and malignant phenotypes in 3D engineered microenvironments. J. Immunother. Cancer.

[35] Shu X., Chen M., Liu S.Y., Yu L., Sun L.X., Sun L.C., Ran Y.L. (2023). Palladin promotes cancer stem cell-like properties in lung cancer by activating Wnt/Β-Catenin signaling. Cancer Med..

[36] Jo M.K., Moon C.M., Kim E.J., Kwon J.H., Fei X., Kim S.E., Jung S.A., Kim M., Mun Y.C., Ahn Y.H. (2022). Suppressive effect of α-mangostin for cancer stem cells in colorectal cancer via the Notch pathway. BMC Cancer.

[37] Wang W., Chen S., Wang Y., Jie C., Shen W. (2025). Effects of Notch signaling on proliferation, angiogenesis, and adipogenesis of hemangioma-derived stem cells. Eur. J. Histochem..

[38] Borlak F., Reutzel-Selke A., Schirmeier A., Gogolok J., von Hoerschelmann E., Sauer I.M., Pratschke J., Bahra M., Schmuck R.B. (2021). Notch Signaling Pathway in Pancreatobiliary Tumors. Medicina.

[39] Zema S., Di Fazio F., Pelullo M., Di Savino S., Cerbelli B., Leopizzi M., Di Magno L., Nicoletti C., Peruzzi G., D’Andrea D. (2026). MAML1 drives Notch and Hedgehog oncogenic pathways by inhibiting Itch activity in triple-negative breast cancer. Cell Death Differ..

[40] Cao L., Weng K., Li L., Lin G., Zhao Y., Gao Y., Huang X., Chen Q., Wang J., Zheng C. (2024). BATF2 inhibits the stem cell-like properties and -chemoresistance of gastric cancer cells through PTEN/AKT/β-catenin pathway. Theranostics.

[41] Giroux Leprieur E., Tolani B., Li H., Leguay F., Hoang N.T., Acevedo L.A., Jin J.Q., Tseng H.H., Yue D., Kim I.J. (2017). Membrane-bound full-length Sonic Hedgehog identifies cancer stem cells in human non-small cell lung cancer. Oncotarget.

[42] Karpova A., Li X., Peng C.W., Gallant K.L., Rapp D.R., Alligood D.M., Houston A.J., Park A., Targino da Costa A.L.N., Chou W.H. (2026). Cellular senescence in human liver under normal aging and cancer. Cell Genom..

[43] Yang J.H., Hayano M., Griffin P.T., Amorim J.A., Bonkowski M.S., Apostolides J.K., Salfati E.L., Blanchette M., Munding E.M., Bhakta M. (2023). Loss of epigenetic information as a cause of mammalian aging. Cell.

[44] Bi Y., Qiao X., Cai Z., Zhao H., Ye R., Liu Q., Gao L., Liu Y., Liang B., Liu Y. (2025). Exosomal miR-302b rejuvenates aging mice by reversing the proliferative arrest of senescent cells. Cell Metab..

[45] Galanos P., Vougas K., Walter D., Polyzos A., Maya-Mendoza A., Haagensen E.J., Kokkalis A., Roumelioti F.M., Gagos S., Tzetis M. (2016). Chronic p53-independent p21 expression causes genomic instability by deregulating replication licensing. Nat. Cell Biol..

[46] Zampetidis C.P., Galanos P., Angelopoulou A., Zhu Y., Polyzou A., Karamitros T., Kotsinas A., Lagopati N., Mourkioti I., Mirzazadeh R. (2021). A recurrent chromosomal inversion suffices for driving escape from oncogene-induced senescence via subTAD reorganization. Mol. Cell.

[47] Beauséjour C.M., Krtolica A., Galimi F., Narita M., Lowe S.W., Yaswen P., Campisi J. (2003). Reversal of human cellular senescence: Roles of the p53 and p16 pathways. EMBO J..

[48] Volonte D., Vyas A.R., Chen C., Dacic S., Stabile L.P., Kurland B.F., Abberbock S.R., Burns T.F., Herman J.G., Di Y.P. (2018). Caveolin-1 promotes the tumor suppressor properties of oncogene-induced cellular senescence. J. Biol. Chem..

[49] Casella G., Munk R., Kim K.M., Piao Y., De S., Abdelmohsen K., Gorospe M. (2019). Transcriptome signature of cellular senescence. Nucleic Acids Res..

[50] Gu Y., Xu T., Fang Y., Shao J., Hu T., Wu X., Shen H., Xu Y., Zhang J., Song Y. (2024). CBX4 counteracts cellular senescence to desensitize gastric cancer cells to chemotherapy by inducing YAP1 SUMOylation. Drug Resist. Updates.

[51] Wallis R., Milligan D., Hughes B., Mizen H., López-Domínguez J.A., Eduputa U., Tyler E.J., Serrano M., Bishop C.L. (2022). Senescence-associated morphological profiles (SAMPs): An image-based phenotypic profiling method for evaluating the inter and intra model heterogeneity of senescence. Aging.

[52] Chen H.A., Ho Y.J., Mezzadra R., Adrover J.M., Smolkin R., Zhu C., Woess K., Bernstein N., Schmitt G., Fong L. (2023). Senescence Rewires Microenvironment Sensing to Facilitate Antitumor Immunity. Cancer Discov..

[53] Lai P., Liu L., Bancaro N., Troiani M., Calì B., Li Y., Chen J., Singh P.K., Alvarez Arzola R., Attanasio G. (2025). Mitochondrial DNA released by senescent tumor cells enhances PMN-MDSC-driven immunosuppression through the cGAS-STING pathway. Immunity.

[54] Laberge R.M., Sun Y., Orjalo A.V., Patil C.K., Freund A., Zhou L., Curran S.C., Davalos A.R., Wilson-Edell K.A., Liu S. (2015). MTOR regulates the pro-tumorigenic senescence-associated secretory phenotype by promoting IL1A translation. Nat. Cell Biol..

[55] Shi T., van Soest D.M.K., Polderman P.E., Burgering B.M.T., Dansen T.B. (2021). DNA damage and oxidant stress activate p53 through differential upstream signaling pathways. Free Radic. Biol. Med..

[56] Yan Y., Gu Y. (2026). Orientin inhibits cyclin E/cyclin A-CDK2 to induce growth arrest at senescence in human gastric cancer cells. Food Chem. Toxicol..

[57] Nassour J., Martien S., Martin N., Deruy E., Tomellini E., Malaquin N., Bouali F., Sabatier L., Wernert N., Pinte S. (2016). Defective DNA single-strand break repair is responsible for senescence and neoplastic escape of epithelial cells. Nat. Commun..

[58] Acevedo M., Vernier M., Mignacca L., Lessard F., Huot G., Moiseeva O., Bourdeau V., Ferbeyre G. (2016). A CDK4/6-dependent epigenetic mechanism protects cancer cells from PML-induced senescence. Cancer Res..

[59] Su W., Hong L., Xu X., Huang S., Herpai D., Li L., Xu Y., Truong L., Hu W.Y., Wu X. (2018). miR-30 disrupts senescence and promotes cancer by targeting both p16INK4A and DNA damage pathways. Oncogene.

[60] Basisty N., Kale A., Jeon O.H., Kuehnemann C., Payne T., Rao C., Holtz A., Shah S., Sharma V., Ferrucci L. (2020). A proteomic atlas of senescence-associated secretomes for aging biomarker development. PLoS Biol..

[61] Liu Y., Feng Y., Cheng L., Xu Y., Wu A., Cheng P. (2025). Profiling with senescence-associated secretory phenotype score identifies GDC-0879 as a small molecule sensitizing glioblastoma to anti-PD1. Cell Death Dis..

[62] Colucci M., Zumerle S., Bressan S., Gianfanti F., Troiani M., Valdata A., D’Ambrosio M., Pasquini E., Varesi A., Cogo F. (2024). Retinoic acid receptor activation reprograms senescence response and enhances anti-tumor activity of natural killer cells. Cancer Cell.

[63] Ruscetti M., Morris J.P., Mezzadra R., Russell J., Leibold J., Romesser P.B., Simon J., Kulick A., Ho Y.J., Fennell M. (2020). Senescence-induced vascular remodeling creates therapeutic vulnerabilities in pancreas cancer. Cell.

[64] Ye J., Baer J.M., Faget D.V., Morikis V.A., Ren Q., Melam A., Delgado A.P., Luo X., Bagchi S.M., Belle J.I. (2024). Senescent CAFs Mediate Immunosuppression and Drive Breast Cancer Progression. Cancer Discov..

[65] Ortiz-Montero P., Londoño-Vallejo A., Vernot J.P. (2017). Senescence-associated IL-6 and IL-8 cytokines induce a self- and cross-reinforced senescence/inflammatory milieu strengthening tumorigenic capabilities in the MCF-7 breast cancer cell line. Cell Commun. Signal..

[66] Li T., Zhang Y., Zhou X., Ji S., Zhou Z., Wang W., Shen L., Liu H., Xie Z., Ruan Y. (2026). Di-n-butyl phthalate induces NF-κB-mediated senescence-associated secretory phenotype to promote epithelial proliferation, epithelial-mesenchymal transition, and benign prostatic hyperplasia. Environ. Int..

[67] Lei Y., Zhong C., Zhang J., Zheng Q., Xu Y., Li Z., Huang C., Ren T. (2025). Senescent lung fibroblasts in idiopathic pulmonary fibrosis facilitate non-small cell lung cancer progression by secreting exosomal MMP1. Oncogene.

[68] Fan G., Yu B., Tang L., Zhu R., Chen J., Zhu Y., Huang H., Zhou L., Liu J., Wang W. (2024). TSPAN8^+^ myofibroblastic cancer-associated fibroblasts promote chemoresistance in patients with breast cancer. Sci. Transl. Med..

[69] Kandhaya-Pillai R., Miro-Mur F., Alijotas-Reig J., Tchkonia T., Kirkland J.L., Schwartz S. (2017). TNFα-senescence initiates a STAT-dependent positive feedback loop, leading to a sustained interferon signature, DNA damage, and cytokine secretion. Aging.

[70] Lim S.O., Li C.W., Xia W., Cha J.H., Chan L.C., Wu Y., Chang S.S., Lin W.C., Hsu J.M., Hsu Y.H. (2016). Deubiquitination and Stabilization of PD-L1 by CSN5. Cancer Cell.

[71] Ostrowska K., Niewinski P., Piotrowski I., Ostapowicz J., Koczot S., Suchorska W.M., Golusiński P., Masternak M.M., Golusiński W. (2024). Senescence in head and neck squamous cell carcinoma: Relationship between senescence-associated secretory phenotype (SASP) mRNA expression level and clinicopathological features. Clin. Transl. Oncol..

[72] Du Y., Cao J., Jiang X., Cai X., Wang B., Wang Y., Wang X., Xue B. (2021). Comprehensive analysis of CXCL12 expression reveals the significance of inflammatory fibroblasts in bladder cancer carcinogenesis and progression. Cancer Cell Int..

[73] Walle T., Kraske J.A., Liao B., Lenoir B., Timke C., von Bohlen Und Halbach E., Tran F., Griebel P., Albrecht D., Ahmed A. (2022). Radiotherapy orchestrates natural killer cell dependent antitumor immune responses through CXCL8. Sci. Adv..

[74] Gu X., Zhu Y., Su J., Wang S., Su X., Ding X., Jiang L., Fei X., Zhang W. (2024). Lactate-induced activation of tumor-associated fibroblasts and IL-8-mediated macrophage recruitment promote lung cancer progression. Redox Biol..

[75] Matsuda S., Revandkar A., Dubash T.D., Ravi A., Wittner B.S., Lin M., Morris R., Burr R., Guo H., Seeger K. (2023). TGF-β in the microenvironment induces a physiologically occurring immune-suppressive senescent state. Cell Rep..

[76] Enomoto Y., Katsura H., Fujimura T., Ogata A., Baba S., Yamaoka A., Kihara M., Abe T., Nishimura O., Kadota M. (2023). Autocrine TGF-β-positive feedback in profibrotic AT2-lineage cells plays a crucial role in non-inflammatory lung fibrogenesis. Nat. Commun..

[77] Tian Q., Wang M., Mubeen F., Sundas A., Naz R., Hu X., Chen X. (2025). CDKN2A and matrix metalloproteinases: Key regulators of cellular senescence in squamous cell carcinoma. Am. J. Transl. Res..

[78] Kavanagh E.L., Lindsay S., Halasz M., Gubbins L.C., Weiner-Gorzel K., Guang M.H.Z., McGoldrick A., Collins E., Henry M., Blanco-Fernández A. (2017). Protein and chemotherapy profiling of extracellular vesicles harvested from therapeutic induced senescent triple negative breast cancer cells. Oncogenesis.

[79] Zhang D., Zhang J.W., Xu H., Chen X., Gao Y., Jiang H.G., Wang Y., Wu H., Yang L., Wang W.B. (2024). Therapy-induced senescent tumor cell-derived extracellular vesicles promote colorectal cancer progression through SERPINE1-mediated NF-κB p65 nuclear translocation. Mol. Cancer.

[80] Uruski P., Sepetowska A., Konieczna C., Pakuła M., Wyrwa M., Tussupkaliyev A., Tykarski A., Mikuła-Pietrasik J., Książek K. (2021). Primary high-grade serous ovarian cancer cells are sensitive to senescence induced by carboplatin and paclitaxel in vitro. Cell. Mol. Biol. Lett..

[81] George N., Joshi M.B., Satyamoorthy K. (2024). DNA damage-induced senescence is associated with metabolomic reprogramming in breast cancer cells. Biochimie.

[82] Maskey R.S., Wang F., Lehman E., Wang Y., Emmanuel N., Zhong W., Jin G., Abraham R.T., Arndt K.T., Myers J.S. (2021). Sustained mTORC1 activity during palbociclib-induced growth arrest triggers senescence in ER+ breast cancer cells. Cell Cycle.

[83] Glück S., Guey B., Gulen M.F., Wolter K., Kang T.W., Schmacke N.A., Bridgeman A., Rehwinkel J., Zender L., Ablasser A. (2017). Innate immune sensing of cytosolic chromatin fragments through cGAS promotes senescence. Nat. Cell Biol..

[84] Pardella E., Pranzini E., Nesi I., Parri M., Spatafora P., Torre E., Muccilli A., Castiglione F., Fambrini M., Sorbi F. (2022). Therapy-induced stromal senescence promoting aggressiveness of prostate and ovarian cancer. Cells.

[85] Iannello A., Thompson T.W., Ardolino M., Lowe S.W., Raulet D.H. (2013). p53-dependent chemokine production by senescent tumor cells supports NKG2D-dependent tumor elimination by natural killer cells. J. Exp. Med..

[86] Majewska J., Agrawal A., Mayo A., Roitman L., Chatterjee R., Sekeresova Kralova J., Landsberger T., Katzenelenbogen Y., Meir-Salame T., Hagai E. (2024). p16-dependent increase of PD-L1 stability regulates immunosurveillance of senescent cells. Nat. Cell Biol..

[87] Hwang H.J., Kang D., Shin J., Jung J., Ko S., Jung K.H., Hong S.S., Park J.E., Oh M.J., An H.J. (2025). Therapy-induced senescent cancer cells contribute to cancer progression by promoting ribophorin 1-dependent PD-L1 upregulation. Nat. Commun..

[88] Bajtai E., Kiss C., Bakos É., Langó T., Lovrics A., Schád É., Tisza V., Hegedűs K., Fürjes P., Szabó Z. (2025). Therapy-induced senescence is a transient drug resistance mechanism in breast cancer. Mol. Cancer.

[89] Chaib S., López-Domínguez J.A., Lalinde-Gutiérrez M., Prats N., Marin I., Boix O., García-Garijo A., Meyer K., Muñoz M.I., Aguilera M. (2024). The efficacy of chemotherapy is limited by intratumoral senescent cells expressing PD-L2. Nat. Cancer.

[90] Yang L., Fang J., Chen J. (2017). Tumor cell senescence response produces aggressive variants. Cell Death Discov..

[91] Milanovic M., Fan D.N.Y., Belenki D., Däbritz J.H.M., Zhao Z., Yu Y., Dörr J.R., Dimitrova L., Lenze D., Monteiro Barbosa I.A. (2018). Senescence-associated reprogramming promotes cancer stemness. Nature.

[92] Karabicici M., Alptekin S., Fırtına Karagonlar Z., Erdal E. (2021). Doxorubicin-induced senescence promotes stemness and tumorigenicity in EpCAM-/CD133- nonstem cell population in hepatocellular carcinoma cell line, HuH-7. Mol. Oncol..

[93] Cahu J., Bustany S., Sola B. (2012). Senescence-associated secretory phenotype favors the emergence of cancer stem-like cells. Cell Death Dis..

[94] Radharani N.N.V., Yadav A.S., Nimma R., Kumar T.V.S., Bulbule A., Chanukuppa V., Kumar D., Patnaik S., Rapole S., Kundu G.C. (2022). Tumor-associated macrophage derived IL-6 enriches cancer stem cell population and promotes breast tumor progression via Stat-3 pathway. Cancer Cell Int..

[95] Jin F., Miao Y., Xu P., Qiu X. (2018). IL-8 regulates the stemness properties of cancer stem cells in the small-cell lung cancer cell line H446. OncoTargets Ther..

[96] Tsubakihara Y., Ohata Y., Okita Y., Younis S., Eriksson J., Sellin M.E., Ren J., Ten Dijke P., Miyazono K., Hikita A. (2022). TGFβ selects for pro-stemness over pro-invasive phenotypes during cancer cell epithelial-mesenchymal transition. Mol. Oncol..

[97] Demaria M., O’Leary M.N., Chang J., Shao L., Liu S., Alimirah F., Koenig K., Le C., Mitin N., Deal A.M. (2017). Cellular Senescence Promotes Adverse Effects of Chemotherapy and Cancer Relapse. Cancer Discov..

[98] Perkins D.W., Steiner I., Haider S., Robertson D., Buus R., O’Leary L., Isacke C.M. (2023). Therapy-induced normal tissue damage promotes breast cancer metastasis. iScience.

[99] Miranda A., Hamilton P.T., Zhang A.W., Pattnaik S., Becht E., Mezheyeuski A., Bruun J., Micke P., de Reynies A., Nelson B.H. (2019). Cancer stemness, intratumoral heterogeneity, and immune response across cancers. Proc. Natl. Acad. Sci. USA.

[100] Zhao W., Li Y., Zhang X. (2017). Stemness-related markers in cancer. Cancer Transl. Med..

[101] Zhang Z., Wang Z.X., Chen Y.X., Wu H.X., Yin L., Zhao Q., Luo H.Y., Zeng Z.L., Qiu M.Z., Xu R.H. (2022). Integrated analysis of single-cell and bulk RNA sequencing data reveals a pan-cancer stemness signature predicting immunotherapy response. Genome Med..

[102] Li X., Wang X., Xie C., Zhu J., Meng Y., Chen Y., Li Y., Jiang Y., Yang X., Wang S. (2018). Sonic hedgehog and Wnt/β-catenin pathways mediate curcumin inhibition of breast cancer stem cells. Anti Cancer Drugs.

[103] Liu H.T., Ho Y.S. (2018). Anticancer effect of curcumin on breast cancer and stem cells. Food Sci. Hum. Wellness.

[104] Hu C., Li M., Guo T., Wang S., Huang W., Yang K., Liao Z., Wang J., Zhang F., Wang H. (2019). Anti-metastasis activity of curcumin against breast cancer via the inhibition of stem cell-like properties and EMT. Phytomedicine.

[105] Huang Y.H., Chiu L.Y., Tseng J.S., Hsu K.H., Chen C.H., Sheu G.T., Yang T.Y. (2024). Attenuation of PI3K-Akt-mTOR pathway to reduce cancer stemness on chemoresistant lung cancer cells by shikonin and synergy with BEZ235 inhibitor. Int. J. Mol. Sci..

[106] Wang H.G., Lu M.X., Sheng M.L., Lou Y.B., Xiao Y.C., Guo A., Yu Z.H. (2025). Shikonin inhibits multiple tumor malignant phenotypes and is associated with Hedgehog pathway downregulation in lung adenocarcinoma. Sci. Rep..

[107] Qian X., Zhu L., Xu M., Liu H., Yu X., Shao Q., Qin J. (2023). Shikonin suppresses small cell lung cancer growth via inducing ATF3-mediated ferroptosis to promote ROS accumulation. Chem. Biol. Interact..

[108] Zhou C., Qian W., Ma J., Cheng L., Jiang Z., Yan B., Li J., Duan W., Sun L., Cao J. (2019). Resveratrol enhances the chemotherapeutic response and reverses the stemness induced by gemcitabine in pancreatic cancer cells via targeting SREBP 1. Cell Prolif..

[109] Davoodvandi A., Darvish M., Borran S., Nejati M., Mazaheri S., Tamtaji O.R., Hamblin M.R., Masoudian N., Mirzaei H. (2020). The therapeutic potential of resveratrol in a mouse model of melanoma lung metastasis. Int. Immunopharmacol..

[110] Song Y., Chen Y., Li Y., Lyu X., Cui J., Cheng Y., Zheng T., Zhao L., Zhao G. (2019). Resveratrol suppresses epithelial-mesenchymal transition in GBM by regulating Smad-dependent signaling. Biomed. Res. Int..

[111] Zhu J., Jiang Y., Yang X., Wang S., Xie C., Li X., Li Y., Chen Y., Wang X., Meng Y. (2017). Wnt/β-catenin pathway mediates (−)-Epigallocatechin-3-gallate (EGCG) inhibition of lung cancer stem cells. Biochem. Biophys. Res. Commun..

[112] Chen Y., Wang X.Q., Zhang Q., Zhu J.Y., Li Y., Xie C.F., Li X.T., Wu J.S., Geng S.S., Zhong C.Y. (2017). (−)-Epigallocatechin-3-gallate inhibits colorectal cancer stem cells by suppressing Wnt/β-catenin pathway. Nutrients.

[113] Naveen C.R., Gaikwad S., Agrawal-Rajput R. (2016). Berberine induces neuronal differentiation through inhibition of cancer stemness and epithelial-mesenchymal transition in neuroblastoma cells. Phytomedicine.

[114] Toden S., Tran H.M., Tovar-Camargo O.A., Okugawa Y., Goel A. (2016). Epigallocatechin-3-gallate targets cancer stem-like cells and enhances 5-fluorouracil chemosensitivity in colorectal cancer. Oncotarget.

[115] Zhao Y., Yang X., Zhao J., Gao M., Zhang M., Shi T., Zhang F., Zheng X., Pan Y., Shao D. (2021). Berberine inhibits chemotherapy-exacerbated ovarian cancer stem cell-like characteristics and metastasis through GLI1. Eur. J. Pharmacol..

[116] El-Benhawy S.A., El-Sheredy H.G., Ghanem H.B., Abo El-Soud A.A. (2020). Berberine can amplify cytotoxic effect of radiotherapy by targeting cancer stem cells. Breast Cancer Manag..

[117] Ibrahim N., Alsadi N., Yasavoli-Sharahi H., Shahbazi R., Hebbo M.J., Kambli D., Balcells F., Matar C. (2025). Berberine inhibits breast cancer stem cell development and decreases inflammation: Involvement of miRNAs and IL-6. Curr. Dev. Nutr..

[118] Tiwari A., Modi S.J., Gabhe S.Y., Kulkarni V.M. (2021). Evaluation of piperine against cancer stem cells (CSCs) of hepatocellular carcinoma: Insights into epithelial-mesenchymal transition (EMT). Bioorg. Chem..

[119] Hakeem A.N., El-Kersh D.M., Hammam O., Elhosseiny A., Zaki A., Kamel K., Yasser L., Barsom M., Ahmed M., Gamal M. (2024). Piperine enhances doxorubicin sensitivity in triple-negative breast cancer by targeting the PI3K/Akt/mTOR pathway and cancer stem cells. Sci. Rep..

[120] Warrier N.M., Krishnan R.K., Prabhu V., Hariharapura R.C., Agarwal P., Kumar P. (2022). Survivin inhibition by piperine sensitizes glioblastoma cancer stem cells and leads to better drug response. Int. J. Mol. Sci..

[121] Varela-Eirín M., Demaria M. (2022). Cellular senescence. Curr. Biol..

[122] Ruhland M.K., Loza A.J., Capietto A.H., Luo X., Knolhoff B.L., Flanagan K.C., Belt B.A., Alspach E., Leahy K., Luo J. (2016). Stromal senescence establishes an immunosuppressive microenvironment that drives tumorigenesis. Nat. Commun..

[123] Lee D.Y., Lee S.J., Chandrasekaran P., Lamichhane G., O’Connell J.F., Egan J.M., Kim Y. (2023). Dietary Curcumin Attenuates Hepatic Cellular Senescence by Suppressing the MAPK/NF-κB Signaling Pathway in Aged Mice. Antioxidants.

[124] Lim J.S., Lee D.Y., Lim J.H., Oh W.K., Park J.T., Park S.C., Cho K.A. (2022). Piperine: An Anticancer and Senostatic Drug. Front. Biosci..

[125] Sanders T.H., McMichael R.W., Hendrix K.W. (2000). Occurrence of resveratrol in edible peanuts. J. Agric. Food Chem..

[126] Liu L., Yang X., Liao Y., Wang C., Wang Y. (2022). Resveratrol alleviates Ang II-induced vascular smooth muscle cell senescence by upregulating E2F1/SOD2 axis. Toxicol. Res..

[127] Zhang Y., Wang T., Wang L., Fang Z., Lu T., Chen S., Yan J., Qin C., Shen X., Kang N. (2025). Epigallocatechin-3-Gallate from Green Tea Reduces Vascular Aging and Endothelial Cell Senescence by Modifying Autophagy and Ferroptosis through the Sirtuin 1 Signaling Pathway. ACS Omega.

[128] Li C., Jiang S., Wang H., Wang Y., Han Y., Jiang J. (2022). Berberine exerts protective effects on cardiac senescence by regulating the Klotho/SIRT1 signaling pathway. Biomed. Pharmacother..

[129] Zheng H., Huang Q., Huang S., Yang X., Zhu T., Wang W., Wang H., He S., Ji L., Wang Y. (2018). Senescence Inducer Shikonin ROS-Dependently Suppressed Lung Cancer Progression. Front. Pharmacol..

[130] Chen J., Li Y., Yu T.S., McKay R.M., Burns D.K., Kernie S.G., Parada L.F. (2012). A restricted cell population propagates glioblastoma growth after chemotherapy. Nature.

[131] Mi Y.J., Liang Y.J., Huang H.B., Zhao H.Y., Wu C.P., Wang F., Tao L.Y., Zhang C.Z., Dai C.L., Tiwari A.K. (2010). Apatinib (YN968D1) reverses multidrug resistance by inhibiting the efflux function of multiple ATP-binding cassette transporters. Cancer Res..

[132] Tang Y.A., Chen Y.F., Bao Y., Mahara S., Yatim S.M.J.M., Oguz G., Lee P.L., Feng M., Cai Y., Tan E.Y. (2018). Hypoxic tumor microenvironment activates GLI2 via HIF-1α and TGF-β2 to promote chemoresistance in colorectal cancer. Proc. Natl. Acad. Sci. USA.

[133] Cheng X., Xu M., Wei J., Cao C. (2026). Cancer drug response and resistance: Molecular mechanisms and combating strategies. Signal Transduct. Target. Ther..

[134] Sen G.S., Mohanty S., Hossain D.M.S., Bhattacharyya S., Banerjee S., Chakraborty J., Saha S., Ray P., Bhattacharjee P., Mandal D. (2011). Curcumin enhances the efficacy of chemotherapy by tailoring p65NFκB-p300 cross-talk in favor of p53-p300 in breast cancer. J. Biol. Chem..

[135] Shakibaei M., Buhrmann C., Kraehe P., Shayan P., Lueders C., Goel A. (2014). Curcumin chemosensitizes 5-fluorouracil resistant MMR-deficient human colon cancer cells in high density cultures. PLoS ONE.

[136] Wang L., Hu R., Dai A. (2022). Curcumin Increased the Sensitivity of Non-Small-Cell Lung Cancer to Cisplatin through the Endoplasmic Reticulum Stress Pathway. Evid. Based Complement. Altern. Med..

[137] Öztürk Y., Günaydın C., Yalçın F., Nazıroğlu M., Braidy N. (2019). Resveratrol Enhances Apoptotic and Oxidant Effects of Paclitaxel through TRPM2 Channel Activation in DBTRG Glioblastoma Cells. Oxid. Med. Cell. Longev..

[138] Xu J., Liu D., Niu H., Zhu G., Xu Y., Ye D., Li J., Zhang Q. (2017). Resveratrol reverses Doxorubicin resistance by inhibiting epithelial-mesenchymal transition (EMT) through modulating PTEN/Akt signaling pathway in gastric cancer. J. Exp. Clin. Cancer Res..

[139] Xie C., Liang C., Wang R., Yi K., Zhou X., Li X., Chen Y., Miao D., Zhong C., Zhu J. (2023). Resveratrol suppresses lung cancer by targeting cancer stem-like cells and regulating tumor microenvironment. J. Nutr. Biochem..

[140] Tang H., Zeng L., Wang J., Zhang X., Ruan Q., Wang J., Cui S., Yang D. (2017). Reversal of 5-fluorouracil resistance by EGCG is mediate by inactivation of TFAP2A/VEGF signaling pathway and down-regulation of MDR-1 and P-gp expression in gastric cancer. Oncotarget.

[141] Kou Y., Tong B., Wu W., Liao X., Zhao M. (2020). Berberine Improves Chemo-Sensitivity to Cisplatin by Enhancing Cell Apoptosis and Repressing PI3K/AKT/mTOR Signaling Pathway in Gastric Cancer. Front. Pharmacol..

[142] Wang J., Kang M., Wen Q., Qin Y.T., Wei Z.X., Xiao J.J., Wang R.S. (2017). Berberine sensitizes nasopharyngeal carcinoma cells to radiation through inhibition of Sp1 and EMT. Oncol. Rep..

[143] Xuan Y., Hu X. (2009). Naturally-occurring shikonin analogues--a class of necroptotic inducers that circumvent cancer drug resistance. Cancer Lett..

[144] Kwak S.Y., Jeong Y.K., Kim B.Y., Lee J.Y., Ahn H.J., Jeong J.H., Kim M.S., Kim J., Han Y.H. (2014). β,β-Dimethylacrylshikonin sensitizes human colon cancer cells to ionizing radiation through the upregulation of reactive oxygen species. Oncol. Lett..

[145] Arozal W., Ramadanty W.T., Louisa M., Satyana R.P.U., Hartono G., Fatrin S., Purbadi S., Estuningtyas A., Instiaty I. (2019). Pharmacokinetic Profile of Curcumin and Nanocurcumin in Plasma, Ovary, and Other Tissues. Drug Res..

[146] Zhou Q.M., Sun Y., Lu Y.Y., Zhang H., Chen Q.L., Su S.B. (2017). Curcumin reduces mitomycin C resistance in breast cancer stem cells by regulating Bcl-2 family-mediated apoptosis. Cancer Cell Int..

[147] Guo Z., Zhang Y., Gong Y., Li G., Pan J., Dou D., Ma K., Cui C., Liu Y., Zhu X. (2025). Antibody functionalized curcuma-derived extracellular vesicles loaded with doxorubicin overcome therapy-induced senescence and enhance chemotherapy. J. Control. Release.

[148] Liskova A., Kubatka P., Samec M., Zubor P., Mlyncek M., Bielik T., Samuel S.M., Zulli A., Kwon T.K., Büsselberg D. (2019). Dietary Phytochemicals Targeting Cancer Stem Cells. Molecules.

[149] Khosravi M.A., Seifert R. (2024). Clinical trials on curcumin in relation to its bioavailability and effect on malignant diseases: Critical analysis. Naunyn Schmiedebergs Arch. Pharmacol..

[150] Choudhari A.S., Mandave P.C., Deshpande M., Ranjekar P., Prakash O. (2020). Phytochemicals in Cancer Treatment: From Preclinical Studies to Clinical Practice. Front. Pharmacol..

[151] Kanai M., Otsuka Y., Otsuka K., Sato M., Nishimura T., Mori Y., Kawaguchi M., Hatano E., Kodama Y., Matsumoto S. (2013). A phase I study investigating the safety and pharmacokinetics of highly bioavailable curcumin (Theracurmin) in cancer patients. Cancer Chemother. Pharmacol..

[152] Bhardwaj R.K., Glaeser H., Becquemont L., Klotz U., Gupta S.K., Fromm M.F. (2002). Piperine, a major constituent of black pepper, inhibits human P-glycoprotein and CYP3A4. J. Pharmacol. Exp. Ther..

[153] Lv Y., Li W., Liao W., Jiang H., Liu Y., Cao J., Lu W., Feng Y. (2024). Nano-Drug Delivery Systems Based on Natural Products. Int. J. Nanomed..

[154] Mundekkad D., Cho W.C. (2022). Nanoparticles in Clinical Translation for Cancer Therapy. Int. J. Mol. Sci..

[155] Zhang P., Xiao Y., Sun X., Lin X., Koo S., Yaremenko A.V., Qin D., Kong N., Farokhzad O.C., Tao W. (2023). Cancer nanomedicine toward clinical translation: Obstacles, opportunities, and future prospects. Med.

[156] Loh J.J., Ma S. (2024). Hallmarks of cancer stemness. Cell Stem Cell.

[157] Song K.X., Wang J.X., Huang D. (2023). Therapy-induced senescent tumor cells in cancer relapse. J. Natl. Cancer Cent..

[158] Howells L.M., Iwuji C.O.O., Irving G.R.B., Barber S., Walter H., Sidat Z., Griffin-Teall N., Singh R., Foreman N., Patel S.R. (2019). Curcumin Combined with FOLFOX Chemotherapy Is Safe and Tolerable in Patients with Metastatic Colorectal Cancer in a Randomized Phase IIa Trial. J. Nutr..

[159] Nguyen A.V., Martinez M., Stamos M.J., Moyer M.P., Planutis K., Hope C., Holcombe R.F. (2009). Results of a phase I pilot clinical trial examining the effect of plant-derived resveratrol and grape powder on Wnt pathway target gene expression in colonic mucosa and colon cancer. Cancer Manag. Res..

[160] Kumar N.B., Pow-Sang J., Egan K.M., Spiess P.E., Dickinson S., Salup R., Helal M., McLarty J., Williams C.R., Schreiber F. (2015). Randomized, Placebo-Controlled Trial of Green Tea Catechins for Prostate Cancer Prevention. Cancer Prev. Res..

[161] Chen Y.X., Gao Q.Y., Zou T.H., Wang B.M., Liu S.D., Sheng J.Q., Ren J.L., Zou X.P., Liu Z.J., Song Y.Y. (2020). Berberine versus placebo for the prevention of recurrence of colorectal adenoma: A multicentre, double-blinded, randomised controlled study. Lancet Gastroenterol. Hepatol..

[162] National Cancer Institute (2024). Curcumin and Piperine in Reducing Inflammation for Ureteral Stent-Induced Symptoms in Patients with Cancer.

